# Dynamic-GLEP: a dynamics-informed deep learning framework for ligand efficacy prediction in representative Class A GPCRs

**DOI:** 10.1093/bib/bbag049

**Published:** 2026-02-12

**Authors:** Zhiyi Chen, Yongxin Hao, Yuhong Su, Hans Ågren, Mingan Chen, Zhehuan Fan, Duanhua Cao, Jiacheng Xiong, Wei Zhang, Jin Liu, Xutong Li, Mingyue Zheng, Xi Cheng, Dingyan Wang, Dan Teng

**Affiliations:** School of Life Sciences, Nanjing University, 163 Xianlin Avenue, Nanjing 210023, China; Drug Discovery and Design Center, State Key Laboratory of Drug Research, Shanghai Institute of Materia Medica, Chinese Academy of Sciences, 555 Zuchongzhi Road, Shanghai 201203, China; Drug Discovery and Design Center, State Key Laboratory of Drug Research, Shanghai Institute of Materia Medica, Chinese Academy of Sciences, 555 Zuchongzhi Road, Shanghai 201203, China; Division of Life Science and Medicine, University of Science and Technology of China, 443 Huangshan Road, Hefei 230026, Anhui, China; Lingang Laboratory, 2380 Hechuan Road, Shanghai 200031, China; Division of X-ray Photon Science, Department of Physics and Astronomy, Uppsala University, Box 516, SE-751 20, Uppsala, Sweden; Drug Discovery and Design Center, State Key Laboratory of Drug Research, Shanghai Institute of Materia Medica, Chinese Academy of Sciences, 555 Zuchongzhi Road, Shanghai 201203, China; School of Physical Science and Technology, ShanghaiTech University, 393 Middle Huaxia Road, Shanghai 201210, China; Drug Discovery and Design Center, State Key Laboratory of Drug Research, Shanghai Institute of Materia Medica, Chinese Academy of Sciences, 555 Zuchongzhi Road, Shanghai 201203, China; University of Chinese Academy of Sciences, No. 19A Yuquan Road, Beijing 100049, China; Drug Discovery and Design Center, State Key Laboratory of Drug Research, Shanghai Institute of Materia Medica, Chinese Academy of Sciences, 555 Zuchongzhi Road, Shanghai 201203, China; Innovation Institute for Artificial Intelligence in Medicine of Zhejiang University, College of Pharmaceutical Sciences, Zhejiang University, 866 Yuhangtang Road, Hangzhou, Zhejiang 310058, China; Drug Discovery and Design Center, State Key Laboratory of Drug Research, Shanghai Institute of Materia Medica, Chinese Academy of Sciences, 555 Zuchongzhi Road, Shanghai 201203, China; University of Chinese Academy of Sciences, No. 19A Yuquan Road, Beijing 100049, China; Drug Discovery and Design Center, State Key Laboratory of Drug Research, Shanghai Institute of Materia Medica, Chinese Academy of Sciences, 555 Zuchongzhi Road, Shanghai 201203, China; University of Chinese Academy of Sciences, No. 19A Yuquan Road, Beijing 100049, China; Drug Discovery and Design Center, State Key Laboratory of Drug Research, Shanghai Institute of Materia Medica, Chinese Academy of Sciences, 555 Zuchongzhi Road, Shanghai 201203, China; Innovation Institute for Artificial Intelligence in Medicine of Zhejiang University, College of Pharmaceutical Sciences, Zhejiang University, 866 Yuhangtang Road, Hangzhou, Zhejiang 310058, China; Drug Discovery and Design Center, State Key Laboratory of Drug Research, Shanghai Institute of Materia Medica, Chinese Academy of Sciences, 555 Zuchongzhi Road, Shanghai 201203, China; University of Chinese Academy of Sciences, No. 19A Yuquan Road, Beijing 100049, China; School of Life Sciences, Nanjing University, 163 Xianlin Avenue, Nanjing 210023, China; Drug Discovery and Design Center, State Key Laboratory of Drug Research, Shanghai Institute of Materia Medica, Chinese Academy of Sciences, 555 Zuchongzhi Road, Shanghai 201203, China; Drug Discovery and Design Center, State Key Laboratory of Drug Research, Shanghai Institute of Materia Medica, Chinese Academy of Sciences, 555 Zuchongzhi Road, Shanghai 201203, China; University of Chinese Academy of Sciences, No. 19A Yuquan Road, Beijing 100049, China; Lingang Laboratory, 2380 Hechuan Road, Shanghai 200031, China; Drug Discovery and Design Center, State Key Laboratory of Drug Research, Shanghai Institute of Materia Medica, Chinese Academy of Sciences, 555 Zuchongzhi Road, Shanghai 201203, China; University of Chinese Academy of Sciences, No. 19A Yuquan Road, Beijing 100049, China

**Keywords:** GPCR ligand efficacy, molecular dynamics, conformational ensembles, deep learning, structure-based drug design, transfer learning

## Abstract

G protein–coupled receptors (GPCRs) represent the largest membrane protein family and remain central targets in drug discovery. Ligand efficacy reflects the ability to modulate receptor conformational states and extends beyond binding affinity to underpin functional selectivity. However, most computational approaches still emphasize affinity prediction, with limited capacity to capture the conformational dynamics driving efficacy. Here, we introduce Dynamic-GLEP, a structure- and mechanism-aware framework that integrates molecular dynamics (MD)–derived conformational ensembles with transfer learning on equivariant graph neural networks. By constructing multi-conformation receptor–ligand complexes and fine-tuning the EquiScore model, Dynamic-GLEP identifies conformation-dependent interaction features to distinguish agonists from nonagonists. Applied to the 5-HT1A receptor, the framework achieved an area under the curve (AUC) of 0.74 in cross-validation and 0.71 on an external Food and Drug Administration (FDA)-related dataset. Comparative analyses showed that Holo-based models are advantageous for scaffold optimization, whereas Apo-derived ensembles provided greater adaptability to chemically diverse ligands. Furthermore, extension to the adenosine A2A receptor yielded high performance (AUC > 0.85), underscoring the method’s robustness and transferability under data-scarce conditions. Collectively, these results highlight Dynamic-GLEP as a reliable and interpretable platform for ligand efficacy prediction in Class A GPCRs, with broad potential to support virtual screening, candidate prioritization, and mechanism-driven drug design.

## Graphical Abstract

**Graphical Abstract:**
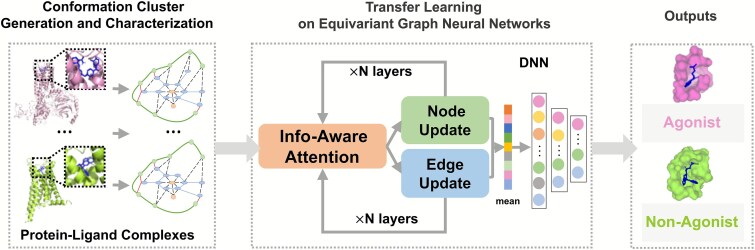

## Introduction

G protein–coupled receptors (GPCRs) represent the largest and most structurally diverse superfamily of transmembrane signaling proteins in the human genome. They play essential roles in neurotransmission, hormone regulation, and sensory perception processes such as vision, olfaction, and taste [1, 2]. These receptors maintain a dynamic equilibrium between active and inactive conformational states, and ligands modulate signaling by selectively stabilizing specific conformations, thereby determining their pharmacological effects. This modulatory capability is generally referred to as efficacy [3–6]. Efficacy is typically quantified by the experimentally measured maximal response (*E*_max_), which reflects a ligand’s intrinsic ability to activate receptor signaling. In this study, we adopt an *E*_max_ threshold of 50% to classify ligands into agonists (*E*_max_ > 50%) and nonagonists (*E*_max_ ≤ 50%). Although *E*_max_ does not further distinguish among nonagonist types, it remains a practical and reproducible index for large-scale agonist screening due to its experimental accessibility.

Developing computational models capable of accurately predicting ligand efficacy during early-stage drug discovery is critical for improving design efficiency and reducing off-target risks. Current computational strategies fall into two major categories: physics-based and machine learning–based approaches [7]. Physics-based methods such as free energy perturbation (FEP) and molecular dynamics (MD) simulations offer atomic level insights into conformational transitions but are often computationally expensive and unsuitable for high-throughput screening [8–11]. In contrast, machine learning models that use ligand or complex structures as input exhibit superior scalability but frequently overlook receptor conformational flexibility. Ligand-based models tend to suffer from limited generalizability due to dataset bias [12, 13], while structure-based models often rely on static conformations or heuristically defined flexible regions, failing to capture ligand-induced structural transitions [14]. Even hybrid models combining ligand and receptor information (e.g. DeepREAL [15]) rarely incorporate the dynamic conformational evolution occurring during binding [16, 17]. Previous studies have demonstrated that ligand efficacy is not solely determined by binding affinity but is also critically dependent on the ability to reshape the receptor’s conformational ensemble [18–23]. Such dynamic effects are difficult to infer from static structures. For example, Power et al. reported that several xanthine derivatives exhibited comparable binding affinities but divergent efficacies toward muscarinic receptor subtypes. MD simulations revealed that this disparity arose from distinct binding modes in the active-state conformations [24, 25], underscoring the limitations of static models in capturing efficacy mechanisms.

To address these challenges, we propose Dynamic GPCR Ligand Efficacy Prediction (Dynamic-GLEP), a deep learning framework that integrates MD simulations with transfer learning to predict GPCR ligand efficacy. The method generates long-timescale MD trajectories and applies a ∆ residue–residue contact score (∆RRCS) [26] strategy to cluster conformations, extracting representative active and inactive receptor states. Ensembles of protein–ligand complexes are then constructed to capture conformation-dependent interaction patterns [27, 28]. Transfer learning is performed on the EquiScore model [29], enabling it to incorporate both equivariant spatial features and ligand-induced conformational dynamics for agonist/nonagonist classification. We evaluated Dynamic-GLEP on two Class A GPCRs: 5-hydroxytryptamine receptor 1A (5-HT1A) and adenosine A2A receptor. The model achieved strong performance and generalization, with area under the curve (AUCs) of 0.74 (5-HT1A test set), 0.71 (external validation set), and 0.85 (A2A test set). Comparative analysis further suggests that holo-state conformations are better suited for lead optimization, whereas apo-state ensembles show greater adaptability in scaffold discovery. Overall, Dynamic-GLEP demonstrates the benefits of integrating conformational dynamics into deep learning–based modeling, offering a new tool and perspective for structure-based GPCR drug discovery.

## Materials and Methods

### Data preparation

#### Training and test set (C-5HT1A)

**Figure 1:**
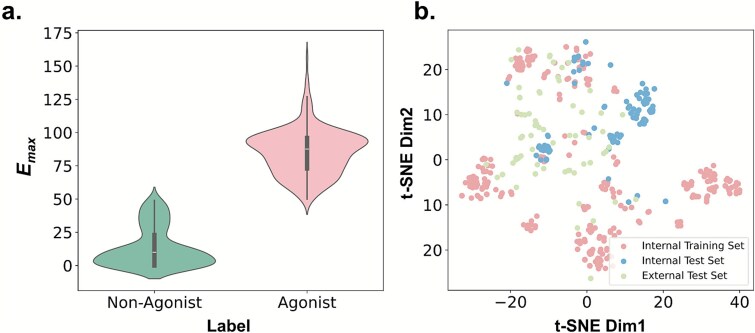
Distribution and t-distributed stochastic neighbor embedding (t-SNE) analysis of C-5HT1A and I-5HT1A ligand datasets. (a) Distribution of *E*_max_ values across two categories of the C-5HT1A ligand dataset. (b) t-SNE analysis of the training and test sets for the C-5HT1A and I-5HT1A datasets.

Curated from ChEMBL 31 (ChEMBL ID: 214, UniProt: P08908) using human 5-HT1A functional assay results, with *E*_max_ (ligand efficacy metric, 50% cutoff for agonists) as the classification basis. After duplicate removal and incomplete entry exclusion, 458 unique compounds were retained (220 agonists, 238 nonagonists; distribution in Fig. 1a). For three-fold cross-validation (fold numbers in Table S1), two splits were used: (i) Tanimoto similarity–based (ECFP4 fingerprints, evaluating novel scaffold performance) and (ii) randomized (assessing baseline accuracy).

#### External test set (I-5HT1A)

The dataset was constructed from 125 IUPHAR-sourced 5-HT1A compounds (including 40 FDA-approved drugs). Following C-5HT1A’s curation (duplicate removal), 73 compounds remained (33 agonists, 40 nonagonists). Dimensionality reduction (Fig. 1b) shows that it occupies distinct chemical space from C-5HT1A, enabling rigorous generalization evaluation.

#### Training and test set (C-A2A)

For A2A (UniProt: P29274), compounds were extracted from ChEMBL251, with processing consistent with C-5HT1A. The final dataset included 75 compounds (22 agonists, 53 nonagonists).

### Generation of protein conformational ensemble

To capture the conformational flexibility of GPCRs associated with ligand efficacy modulation, we performed all-atom MD simulations. Taking 5-HT1A as an example, we constructed both Holo and Apo systems of the 5-HT1A receptor for comparative analysis. For the 5-HT1A Holo system, the initial active-state structure was derived from the 5-HT1A–Gi protein complex (PDB ID: 7E2Z) [30]. The corresponding inactive-state model was generated via homology modeling using Modeller, with the 5-HT2B receptor (PDB ID: 5 V54) serving as the template. Both active and inactive structures were embedded in a 1-palmitoyl-2-oleoyl-glycero-3-phosphocholine (POPC) lipid bilayer using CHARMM-GUI [31], solvated in a cubic TIP3P water box (160 Å × 160 Å × 160 Å), and neutralized with 0.15 M NaCl. MD simulations were performed using GROMACS 2022 [32] with the CHARMM36m all-atom force field [33]. Each system was subjected to a 10-ns equilibration phase followed by a 500-ns production run, conducted in triplicate to ensure statistical robustness. System stability was evaluated across the three replicates by monitoring root-mean-square deviation (RMSD) and key interhelical distances. The replicate showing the most stable RMSD profile was selected for downstream analysis. Simulations were carried out in the isobaric-isothermal (NPT) ensemble at 303.15 K and 1 atm, employing a velocity-rescaling thermostat [33] and the Parrinello–Rahman barostat with isotropic pressure coupling [34]. Nonbonded interactions were treated using a 1.2 nm cutoff for both Lennard–Jones potentials and real-space electrostatics, with long-range electrostatics computed via the Particle Mesh Ewald method [35, 36]. Bond constraints were applied using the linear constraint solver (LINCS) algorithm, and a 2-fs integration time step was used throughout. From three independent 500-ns production trajectories, snapshots were extracted every 1 ns from the final 200 ns of each run and combined into a candidate conformational ensemble. To ensure structural stability and diversity, frames were filtered based on backbone RMSD convergence, pocket volume consistency, and the ΔRRCS to remove redundant or collapsed conformations. A total of 200 representative structures were retained for each receptor form (200 active and 200 inactive), providing a diverse yet physically plausible ensemble for subsequent docking and learning analyses.

For the 5-HT1A Apo system, simulations were initiated from the inactive-state crystal structure. The active-state model was constructed via homology modeling using the 5-HT2B receptor as the template. For the A2A receptor system, the agonist-bound active-state and antagonist-bound inactive-state structures were used directly as initial models. All protein structures were embedded in a POPC lipid bilayer and solvated in a TIP3P water box (160 Å × 160 Å × 160 Å), with 0.15 M NaCl added to neutralize the system. Subsequent MD simulations followed the same equilibration, production, and analysis protocols as described for the 5-HT1A Holo system, ensuring methodological consistency across all receptor systems.

### Principal component analysis of receptor conformational ensemble

To characterize the conformational space sampled during the simulations, PCA was performed for the active and inactive 5-HT1A receptor ensembles using the Cartesian coordinates of all protein Cα atoms. For each state, 200 conformations were uniformly extracted from the equilibrated MD trajectories. All frames were rigidly aligned to state-specific reference structures based on Cα atoms to remove overall translation and rotation. The aligned coordinates were concatenated into high-dimensional data matrices (*n* × 3 *N*), where *n* is the number of frames and *N* is the number of Cα atoms.

**Figure 2:**
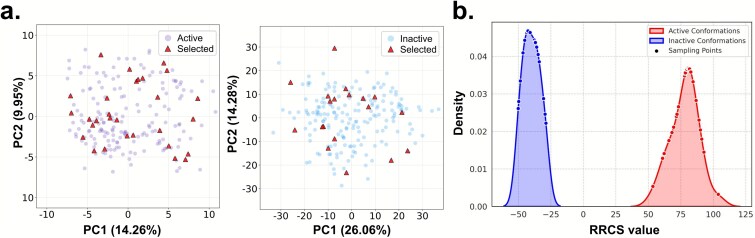
PCA-based visualization and ∆RRCS-based selection of representative 5-HT1A conformations. (a) PC1–PC2 projections of active (left) and inactive (right) MD ensembles; red triangles indicate ∆RRCS-selected representative structures. (b) Distribution of active (right) and inactive (left) conformations and the location of sampled points within the conformational space.

PCA was carried out independently for the active and inactive ensembles using scikit-learn after mean-centering the data. The first two principal components (PC1 and PC2), representing the dominant collective motions, were retained for analysis, and their EVRs were used to quantify the contribution of each mode to the overall structural fluctuations. Projections onto the PC1–PC2 subspaces were used for visualization (Fig. 2a). ΔRRCS-selected representative conformations were mapped onto the corresponding PCA spaces and highlighted to assess their coverage of the underlying conformational distributions. All analyses were performed using MDAnalysis, NumPy, pandas, scikit-learn, and matplotlib.

### Identification of representative conformations via ∆RRCS algorithm

To identify representative conformations that effectively capture the dynamic range of receptor states, we employed the ∆RRCS algorithm, a method designed to quantify residue contact changes across conformational states of GPCRs. Specifically, we targeted four residue pairs (3 × 43, 7 × 49, 3 × 43:7 × 53, 3 × 46:7 × 53, 3 × 50:7 × 53), which are critical for activation in Class A GPCRs, as defined by the Ballesteros–Weinstein numbering system [37].

The ∆RRCS values were normalized to the initial activated conformation for both the 5-HT1A and A2A receptors. Residues with ∆RRCS values above 1 were classified as favoring the active conformation, while values below 1 indicated a preference for the inactive state. This approach allowed us to quantify and distinguish key conformational changes that reflect receptor activation. From the 400 MD snapshots generated for each receptor system, we selected 48 conformations for the 5-HT1A Holo, 60 for the 5-HT1A Apo, and 47 for the A2A receptor, each corresponding to unique, nonredundant snapshots that represent both the active and inactive receptor states. These selected conformations were subsequently used for ensemble docking and further analysis. This method ensures that the selected receptor conformations effectively represent the thermodynamic landscape, balancing the exploration of both the active and inactive states while minimizing redundancy.

### Construction of conformational ensemble for protein–ligand complexes

The selected representative protein conformations were prepared using Schrödinger Maestro (2022-4), specifically employing the Protein Preparation Wizard [38]. This process involved optimizing hydrogen atoms, assigning bond orders, and refining hydrogen bond networks. Missing side chains and loops were reconstructed, and water molecules beyond 5 Å of any ligand were excluded. Following this, energy minimization was performed using the OPLS4 force field [39] until the RMSD of heavy atoms converged to 0.30 Å. For ensemble docking, ligands were prepared using LigPrep with the Epik module to predict possible ionization states at a target pH of 7.0 ± 2.0. The OPLS4 force field was applied to generate ligand conformations, which were then used as starting points for docking. Receptor grids were generated using the Receptor Grid Generation module, with dimensions adjusted to fit the cocrystallized ligand. All prepared protein conformations were subjected to global docking using the standard precision mode of Glide, with the resulting protein–ligand complexes used as inputs for our method.

### Transfer learning framework based on the EquiScore model

**Figure 3:**
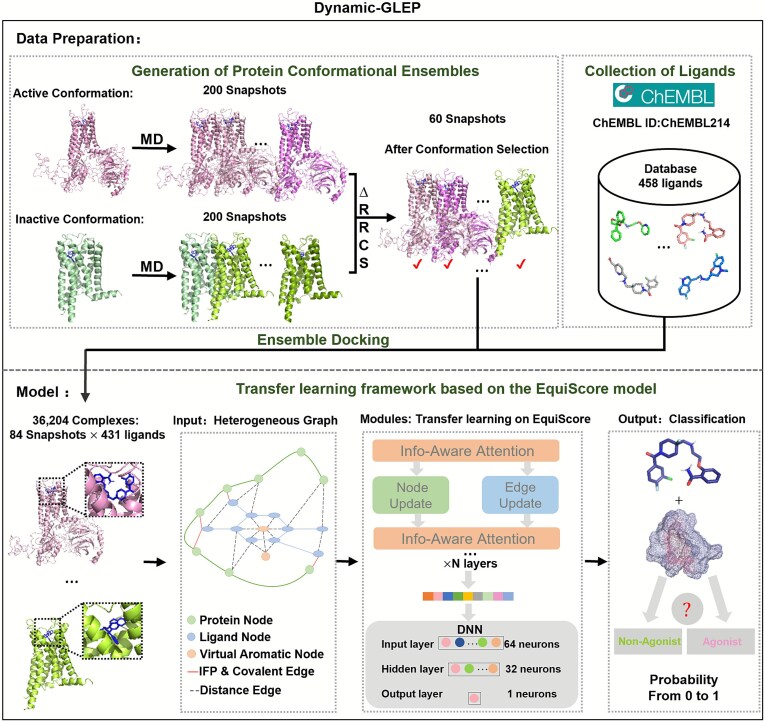
The pipeline of dynamic-GLEP.

To enhance ligand efficacy prediction, we fine-tuned EquiScore (an equivariant graph neural network) via TL. This framework integrates physical domain knowledge to characterize protein–ligand interactions in an equivariant geometric space, boosting robustness and accuracy for GPCR ligand efficacy prediction. The input is a heterogeneous graph derived from the protein’s pocket region and the ligand molecule. This graph is denoted as $G=\left(V,{E}_{\mathrm{geometric}},{E}_{\mathrm{structural}}\right)$. $V=\left\{\ {v}_1,{v}_2,{v}_3,\dots, {v}_n\right\}$ is a set of nodes, where $n$ is the number of the node. Following the EquiScore framework, three node types are included: protein nodes, ligand-atom nodes, and virtual aromatic nodes. Each virtual aromatic node is defined as the centroid of an aromatic ring, enabling explicit representation of π–π and cation–π interactions. ${E}_{\mathrm{geometric}}=\left\{{e}_1,{e}_2,{e}_3,\dots, {e}_{\mathrm{m}}\right\}$ encompasses edges derived from geometric distances, and $m$ denotes the number of edges in ${E}_{\mathrm{geometric}}$. This also includes protein–protein adjacency edges (depicted as green edges in Fig. 3), defined between residues whose ${C}_{\mathrm{\alpha}}$ atoms lie within a distance cutoff, representing intraprotein structural connectivity. ${E}_{\mathrm{structural}}=\left\{{e}_1,{e}_2,{e}_3,\dots, {e}_{\mathrm{k}}\right\}$ comprises edges established on covalent bonds or Interaction Fingerprints (IFP) information, with $k$ indicating the number of edges in ${E}_{\mathrm{structural}}$. Each node $\left({v}_{\mathrm{i}}\right)$ or edge $\left({e}_{\mathrm{i}}\right)$ has a vector ${h}_{\mathrm{i}}$ or ${m}_{\mathrm{i}}$, respectively, representing its contextual information. Node and edge features are mapped into continuous learnable vector representations through embedding layers, following the original EquiScore framework, before being processed by the equivariant message-passing modules.

Graph node and edge representations were initialized via embedding layers, then processed by the EquiScore layer (three submodules: information-aware attention, node update, edge update). The information-aware attention module uses Transformer-based self-attention; node updates were implemented via Equivariant Graph Neural Network [29]:


(1) \begin{equation*} {m}_{ij}={\varnothing}_e\left({h}_i^t,{h}_j^t,\left|\left|{x}_i^t-{x}_j^t\right|\right|,{a}_{ij}\right) \end{equation*}

(2) \begin{equation*} {\displaystyle \begin{array}{c}{x}_i^{t+1}={x}_i^t+\sum_{j\ne i}\left({x}_i^t-{x}_j^t\right){\varnothing}_x\left({m}_{ij}\right)\end{array}} \end{equation*}

(3) \begin{equation*} {\displaystyle \begin{array}{c}{m}_i=\sum_{j\in{N}_{(i)}}{m}_{ij}\end{array}} \end{equation*}

(4) \begin{equation*} {\displaystyle \begin{array}{c}{h}_i^{t+1}={\varnothing}_h\left({h}_i^t,{m}_i\right)\end{array}} \end{equation*}

Here, ${a}_{ij}$ signifies edge features, and *x* denotes coordinates, while ${\varnothing}_{\mathrm{e}},\kern0.5em {\varnothing}_{\mathrm{x}}$, and ${\varnothing}_{\mathrm{h}}$ represent three functions, involving MLPs, activation function, or residual connections. Finally, attention matrices were retrieved by two projection modules and projected into a hidden space with dimensions matching those of edge features to update edges.

Specifically, we used the EquiScore penultimate layer output (high-dimensional complex representations) as input features. After feature extraction, we concatenated multi-conformation representations and fed them into a prediction model consisting of three fully connected layers: the first layer had 64 neurons with Rectified Linear Unit (ReLU) activation for nonlinearity, the second layer had 32 neurons also using ReLU activation, and the final output layer had 1 neuron with Sigmoid activation for agonist and nonagonist binary classification of ligand efficacy. Training adopted the binary cross-entropy loss function, using the Adam optimizer with a batch size of 32 over 25 epochs. Five experimental repetitions were conducted to ensure result stability, and all experiments were performed on the NVIDIA GeForce RTX 3060 graphics processing unit.

### Baseline models

To assess Dynamic-GLEP’s performance, we compared it with eight baseline models, divided into static and dynamic structure–based groups. Static models included Glide_active, Glide_inactive, Glide_TwoStates, EquiScore_active, EquiScore_inactive, and EquiScore_TwoStates—relying on docking scores from single structural snapshots (active/inactive) or active–inactive score differences. Dynamic models were Glide_RF and EquiScore_RF, which use docking scores across MD-generated conformations and are classified via Random Forest (RF) [40].

Static models: Glide_active/EquiScore_active used the first active-state snapshot for docking scoring; Glide_inactive/EquiScore_inactive used the first inactive-state snapshot; and Glide_TwoStates/EquiScore_TwoStates classified via active–inactive score differences. These models rely on static structures, limiting capture of GPCR dynamic binding. Dynamic models use full MD-derived conformation sets and RF to interpret ensemble docking scores—offering a more flexible approach but facing feature extraction and generalization limitations.

### Evaluation metrics

We used five standard classification metrics to rigorously evaluate Dynamic-GLEP’s performance: AUC, ACC, MCC, Precision, and Recall (recall rate), where AUC assesses model ranking ability.

The equations are formulated as follows:


(5) \begin{equation*} {\displaystyle \begin{array}{c} AUC={\int\limits_0^1} True- Positive\ Rate(TPR)d\left(\mathrm{False}-\mathrm{Positive}\ \mathrm{Rate}\right)\end{array}} \end{equation*}

(6) \begin{equation*} {\displaystyle \begin{array}{c} ACC=\frac{TP+ TN}{TP+ FP+ TN+ FN}\end{array}} \end{equation*}

(7) \begin{equation*} {\displaystyle \begin{array}{c} MCC=\frac{TP\times TN- FP\times FN}{\sqrt{\left( TP+ FP\right)\left( TP+ FN\right)\left( TN+ FP\right)\left( TN+ FN\right)}}\end{array}} \end{equation*}

(8) \begin{equation*} {\displaystyle \begin{array}{c} Precision=\frac{TP}{TP+ FP}\end{array}} \end{equation*}

(9) \begin{equation*} {\displaystyle \begin{array}{c} Recall=\frac{TP}{TP+ FN}\end{array}} \end{equation*}

where TP—true-positive count, TN—true-negative count, FP—false-positive count, and FN—false-negative count. Higher values of these metrics indicate better classification performance, with AUC being particularly useful for assessing the model’s ability to distinguish between different ligand efficacy classes.

## Results and discussion

### Overview of the dynamic-GLEP workflow

(1) Generation of receptor conformational ensembles

Five-hundred-nanosecond all-atom MD simulations starting from active and inactive 5-HT1A crystal structures sampled comprehensive conformational landscapes, capturing ligand-induced transitions and activation associated dynamics (Fig. 3).

(2) Representative conformation identification via ∆RRCS

The ∆RRCS strategy quantified contact strength evolution across MD trajectories, enabling efficient clustering of metastable states while preserving thermodynamic relevance and conformational diversity.

(3) Ligand dataset curation and efficacy labeling

5-HT1A ligands from ChEMBL [41] and IUPHAR [42] were labeled by experimental *E*_max_: agonists (*E*_max_ > 50%) and nonagonists (*E*_max_ ≤ 50%). Training (C-5HT1A) and external test (I-5HT1A) datasets with diverse scaffolds ensured broad chemical space coverage.

(4) Construction of protein–ligand complex ensembles

Ensemble docking using ∆RRCS-selected conformations generated diverse protein–ligand complexes, covering multiple states and binding modes to support conformation-dependent interaction modeling.

(5) Transfer learning on EquiScore-based architecture

The EquiScore equivariant graph neural network, which encodes rotation- and translation-invariant 3D features, was transfer-learned on multi-conformational complexes, capturing dynamic efficacy-related interactions for accurate agonist/nonagonist classification.

### Conformational ensemble docking for ligand efficacy assessment

**Figure 4:**
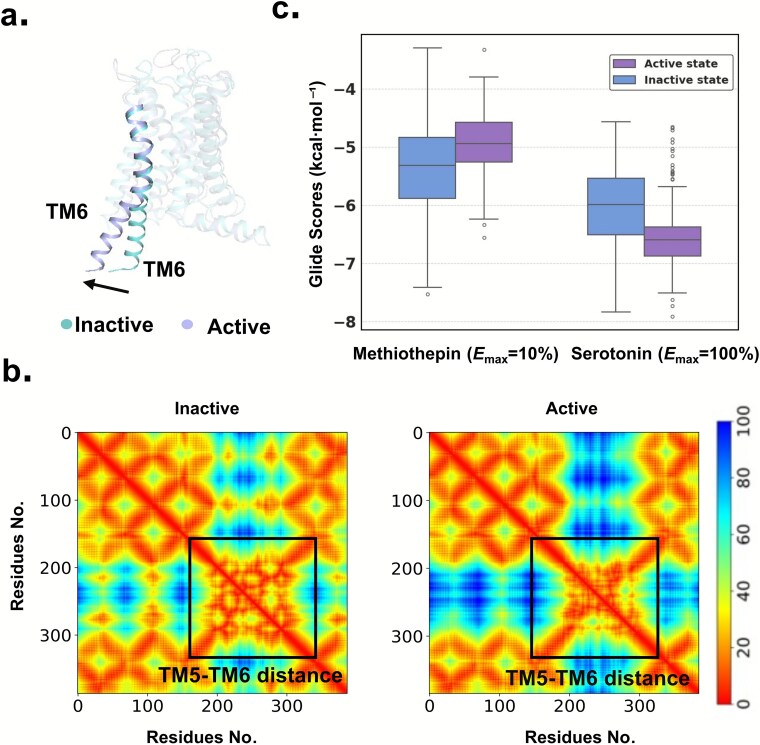
Comparison of GPCR active and inactive conformational states and docking score distributions of representative ligands. (a) Comparison of the position of the sixth transmembrane helix (TM6) between the active state and inactive state. (b) Interhelical contact maps of the inactive (left) and active (right) conformations, illustrating ${C}_{\mathrm{\alpha}}$–${C}_{\mathrm{\alpha}}$ distances between residues in TM5 and TM6. The color scale (0–100 Å) represents interhelical distances, where close packing is shown at the lower end of the scale and TM6 displacement at the higher end. (c) Docking score distributions of representative ligands. The left panel shows the nonagonist methiothepin (*E*_max_ = 10%), and the right panel shows the agonist serotonin (*E*_max_ = 100%). In each panel, the left box corresponds to inactive-state conformations, while the right box corresponds to active-state conformations.

Recent structural biology and molecular simulation studies show that GPCRs exist as dynamic conformational ensembles spanning active, inactive and intermediate states [43, 44], rather than two discrete states. Ligands modulate activity by stabilizing specific conformations; a key GPCR activation hallmark is TM6 outward movement (Fig. 4a and b), which, together with the C-terminal α5-helix of the Gα subunit, reshapes the intracellular cavity to enable G-protein coupling and downstream signaling [45–48]. Thus, a ligand’s efficacy depends on its ability to stabilize specific conformations.

To test this and evaluate if multi-conformation docking scores reflect ligand-modulated conformational distribution, we analyzed two 5-HT1A ligands with distinct *E*_max_: nonagonist methiothepin (*E*_max_ = 10%) and full agonist serotonin (*E*_max_ = 100%). Each was docked to 400 MD-derived receptor snapshots, and binding score distributions across conformations were analyzed. As shown in Fig. 4c, methiothepin had lower (better) docking scores in inactive states (average − 5.36 ± 0.79 kcal·mol^−1^) than in active states (−4.95 ± 0.55 kcal·mol^−1^), consistent with nonagonist function. Conversely, serotonin had lower scores in active states (average − 6.53 ± 0.55 kcal·mol^−1^) than in inactive states (−6.02 ± 0.66 kcal·mol^−1^), supporting full agonist function. Both ligands showed significant statistical differences (*P* < .05) in docking scores between preferred and nonpreferred states.

While only two representative ligands were analyzed here, their opposite conformational preferences provide an illustrative example linking receptor state to efficacy. Together, these observations suggest that ligand efficacy is associated with its ability to modulate receptor conformational distributions. By integrating MD-derived ensembles with docking, Dynamic-GLEP effectively captures these conformation-dependent structural determinants of efficacy, providing a foundation for rational GPCR drug design.

### Selection of representative conformations from the ensemble

To evaluate the structural diversity of the conformational ensembles, principal component analysis (PCA) was performed on 400 uniformly sampled conformations from the active and inactive MD trajectories of 5-HT1A, using high-dimensional backbone structural descriptors derived from protein Cα coordinates. As shown in Fig. 2a, each ensemble was projected onto the 2D subspace spanned by the first two principal components (PC1 and PC2). In both functional states, the PC1–PC2 projections exhibit broadly distributed point clouds rather than compact clusters, indicating substantial intrinsic conformational heterogeneity within each ensemble. For the active state, PC1 and PC2 explain 14.26% and 9.95% of the total variance, respectively, whereas for the inactive state, the corresponding explained variance ratios (EVRs) increase to 26.06% and 14.28%. The higher EVRs observed for the inactive ensemble indicate that its conformational variability is dominated by a smaller number of collective motions, consistent with a more constrained and energetically stable inactive basin, whereas the lower EVRs of the active ensemble reflect a broader distribution of structural fluctuations associated with activation-related flexibility. These results indicate that the dominant conformational variability of 5-HT1A is distributed across multiple collective modes, consistent with the high-dimensional and flexible nature of GPCR dynamics.

To reduce redundancy and enhance representative conformations’ functional relevance, we introduced the ∆RRCS strategy. This method quantifies structural rearrangement via dynamic interconformational contact changes in MD trajectories; it is a transition-sensitive filtering metric with thermodynamic indications, enabling effective identification of key conformations across active, inactive, and intermediate states.

Mapping ∆RRCS-selected conformations to conformational space (Fig. 2b) revealed coverage of high- and low-density regions of the conformational energy landscape (including active, inactive, and transition states), demonstrating good representativeness and diversity. Compared to conventional random sampling, ∆RRCS better captures functional state-switching-related structural changes (e.g. TM6 outward movement, G-protein binding pocket opening), providing a more interpretable training foundation for deep models to learn conformation–efficacy relationships.

In short, mechanistic-driven ∆RRCS extracts key receptor states from high-dimensional MD trajectories. The resulting high-quality data improve the generalization and interpretability of models that predict protein–ligand binding modes and ligand efficacy.

### Ligand efficacy prediction via conformational ensemble and transfer learning

**Figure 5:**
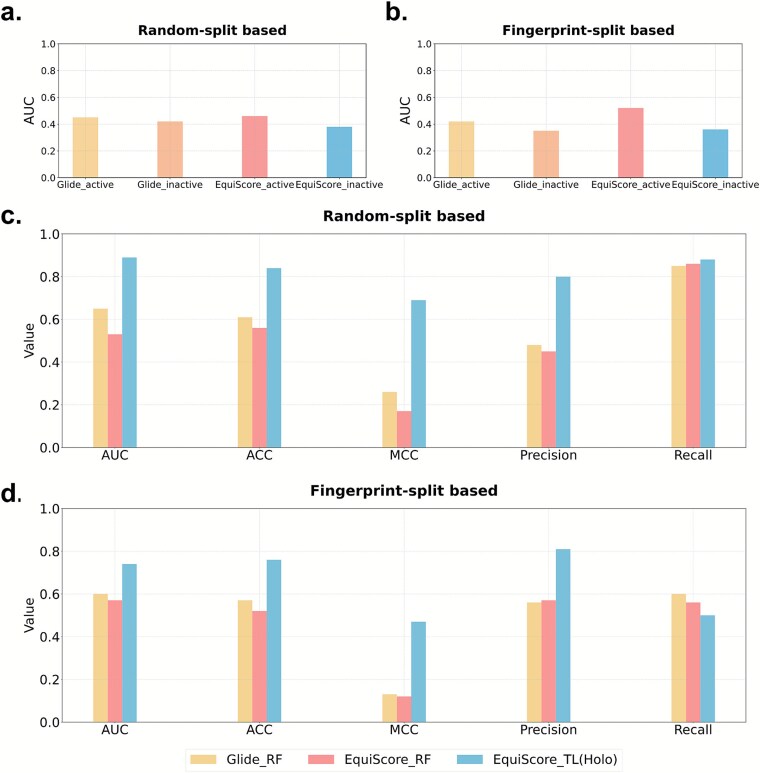
Comparative performance of various LEP methods on the test set. (a) Performance comparison of static crystal structure–based scoring models on a random split. (b) Performance comparison of static crystal structure–based scoring models on a structural similarity–based split. (c) Performance comparison of dynamic conformational ensemble–based models on a random split. (d) Performance comparison of dynamic conformational ensemble–based models on a structural similarity–based split.

To systematically evaluate dynamic conformational sampling and transfer learning in ligand efficacy prediction (LEP), we compared the Dynamic-GLEP method with benchmark models, including static structure-based approaches and dynamic ensemble–incorporating strategies, as detailed in the Methods section. Models based on single crystal structures, including Glide_active, Glide_inactive, EquiScore_active, and EquiScore_inactive, as well as those using two-conformation inputs such as Glide_TwoStates and EquiScore_TwoStates, showed poor performance under both random and structural similarity splits, with AUC values close to 0.5 (Fig. 5a and b), indicating that static snapshots are insufficient to capture the conformational equilibrium features of GPCRs.

In contrast, incorporating MD-sampled conformational ensembles markedly improved model performance (AUC ≈ 0.8; Fig. 5c and d), confirming the essential role of receptor conformational dynamics in efficacy prediction. Among these models, the transfer learning fine-tuned EquiScore_TL achieved an AUC of 0.74, outperforming the nontransfer learning baselines EquiScore_Base (AUC = 0.57) and EquiScore_RF (AUC = 0.64). Under the structural similarity–based split, which evaluates generalization to novel scaffolds, this performance gap highlights the advantage of pretrained equivariant geometric descriptors in capturing conformation-dependent interaction patterns, an ability lacking in traditional scoring functions and architectures without transfer learning. Notably, EquiScore_TL maintained stable performance across splits, whereas benchmark models exhibited pronounced performance drops under structural similarity splits, suggesting that EquiScore_TL captures interaction patterns rather than relying on label memorization. On external validation, Dynamic-GLEP achieved the highest AUC, MCC (Matthews correlation coefficient), ACC (accuracy), and Precision (precision rate), with particularly strong recognition of agonists, consistent with the biological interpretation of *E*_max_. Nonagonists were more difficult to identify, likely due to their greater chemical and mechanistic heterogeneity, which results in diverse and less predictable receptor-binding conformations. This observation underscores a general challenge in efficacy modeling, where inhibitory or inverse ligands often exhibit multiple binding and signaling mechanisms. Incorporating multi-label classification or conformation state–aware learning schemes in future work may further enhance the model’s ability to distinguish such heterogeneous efficacy states.

In conclusion, EquiScore_TL integrates MD and transfer learning to improve the prediction of GPCR ligand efficacy. Future optimizations could enable the fine-grained classification of drug types, aiding high-throughput prediction and functional studies.

### Generalizability assessment of Dynamic-GLEP on an external test set

**Figure 6:**
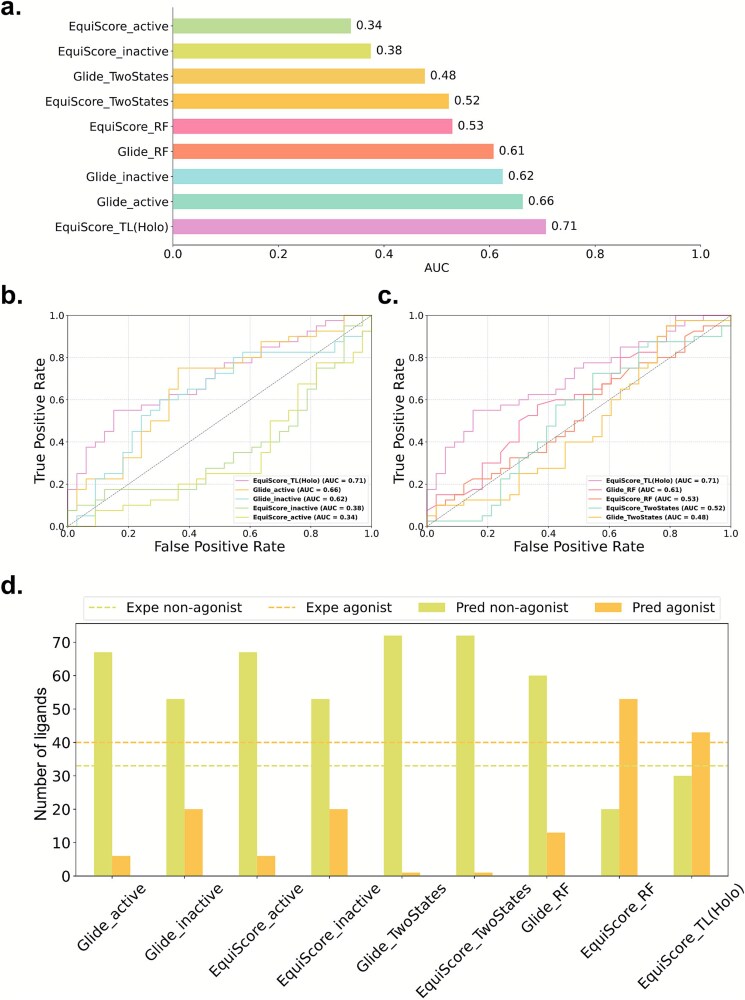
ROC curves and AUC performance on the external test set. (a) Overall performance of all evaluated models on the IUPHAR external dataset. (b) ROC curves comparing EquiScore_TL with static single-structure models. (c) ROC curves comparing EquiScore_TL with dynamic-ensemble models. (d). Comparative statistics of predicted labels versus experimental labels.

To systematically evaluate the generalizability of Dynamic-GLEP, we used the IUPHAR database [45] for external validation. This database contains a diverse range of experimentally validated 5-HT1A ligands and Food and Drug Administration (FDA)-approved drugs, covering various chemical scaffolds and efficacy types. As such, it is highly representative and challenging. The evaluation aimed to assess the model’s ability to predict the efficacy of structurally novel and clinically relevant ligands. On this external dataset, EquiScore_TL demonstrated the best performance, achieving an AUC of 0.71 (Fig. 6a), which was significantly superior to the other baseline models. Notably, models that integrated dynamic conformational information (e.g. EquiScore_RF and Glide_RF) consistently outperformed traditional methods based solely on single static structures, underscoring the critical role of conformational diversity in GPCR ligand efficacy modeling.

A further comparison showed that EquiScore_TL had stronger predictive power than the nonfine-tuned EquiScore_RF, indicating that training with transfer learning on a dataset with true efficacy labels can significantly enhance a model’s ability to recognize functional conformational features. Figure 6b and c further compares EquiScore_TL with static single-structure models and dynamic-ensemble models, respectively, on the external test set. In both comparisons, EquiScore_TL achieved higher AUC values and more favorable receiver operating characteristic (ROC) curves, illustrating the benefit of combining MD-derived conformational ensembles with efficacy-supervised fine-tuning. We also compared the consistency between the model’s predicted labels and the experimental labels (Fig. 6d). The results show that methods incorporating MD conformational information had a higher matching rate with experimental data, whereas traditional static structure methods showed significant deviations on several samples. This result further supports the effectiveness and generalizability of the Dynamic-GLEP framework in real-world application scenarios.

Dynamic-GLEP exhibited the best predictive accuracy in distinguishing between agonists and nonagonists, demonstrating that it not only recognizes key structural features but also successfully models protein–ligand interaction patterns that are closely related to ligand efficacy. It is worth emphasizing that the model maintained a high prediction accuracy even on the more chemically diverse external test set. A further Tanimoto similarity analysis revealed that most ligands in the external test set had low structural similarity to the training set samples, which suggests that EquiScore_TL did not overfit and instead successfully identified generalizable structural features that determine efficacy.

In summary, the external validation results fully demonstrate that Dynamic-GLEP is a robust and generalizable ligand efficacy prediction framework. It is suitable for a wide range of structurally diverse candidate compounds and shows practical utility in GPCR drug screening and hit prioritization.

### Interpretability analysis: case studies of representative ligands

**Figure 7:**
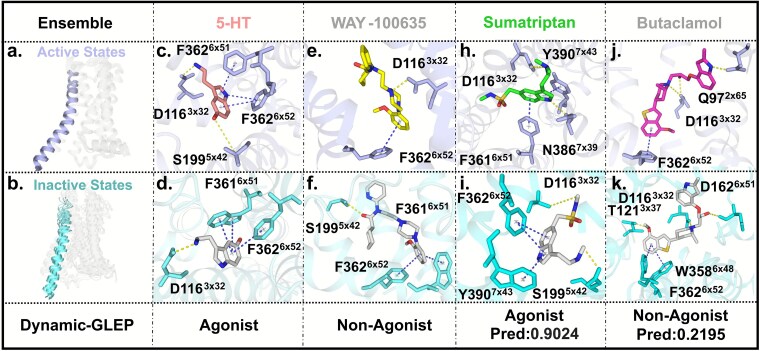
Interpretability analysis of representative 5-HT₁A ligand binding modes predicted by Dynamic-GLEP. (a, b) Active (a) and inactive (b) receptor conformational ensembles highlighting TM5–TM6 rearrangements associated with activation. (c–k) Predicted active and inactive binding modes of 5-HT, WAY-100635, sumatriptan, and butaclamol. Agonists exhibit deeper insertion and active-like hydrogen-bonding patterns, whereas antagonists favor inactive conformations.

To assess the interpretability of Dynamic-GLEP and verify that its predictions are driven by structurally and functionally meaningful protein–ligand interactions rather than ligand scaffold similarity, we analyzed four structurally diverse 5-HT1A ligands (Tanimoto similarity <0.3) representing distinct efficacy classes. Structural comparison of the receptor conformational ensemble showed active/inactive state differences in key regions, especially TM6, which enables G-protein coupling (Fig. 7a and b).

We then analyzed representative ligands’ binding modes across conformational states. Endogenous full agonist serotonin (5-HT) preferentially binds the active state, forming H-bonds with D116^3.32^/S199^5.42^ and π–π interactions with F361^6.51^/F362^6.52^ (Fig. 7c and d) to stabilize the active conformation. Known antagonist WAY-100635 favors the inactive state, stabilizing it via H-bond with D116^3.32^ and π–π interaction with F362^6.52^ (Fig. 7e and f).

In the external test set, the agonist Sumatriptan exhibits clear state-dependent binding behavior. In the active conformation, it forms H-bonds with D116^3.32^ and N386^7.39^, together with π–π interactions involving F361^6.51^ and Y390^7.43^ (Fig. 7h). In contrast, in the inactive state, Sumatriptan engages D116^3.32^ and S199^5.42^ via H-bonds and interacts with F362^6.52^/Y390^7.43^ (Fig. 7i). Comparison of 5-HT binding across receptor states further illustrates distinct interaction geometries associated with efficacy (Fig. 7c and d). In the active state, 5-HT penetrates deeper toward the TM5–TM6 interface, forming a stable hydrogen bond with D116^3.32^ and an additional polar contact with S199^5.42^. In the inactive state, the ligand adopts a higher pocket position, the interaction with D116^3.32^ weakens, and TM5 contacts are lost, consistent with an inactive receptor configuration. By contrast, the nonagonist WAY-100635 preferentially stabilizes the inactive state through a hydrogen bond with S199^5.42^ and π–π interactions with F362^6.52^, potentially contributing to receptor inactivation. Butaclamol and Robalzotan display binding preferences consistent with their experimentally characterized functional profiles (Fig. 7j and k; detailed modes in the Supporting Information).

These structure–function comparative analyses indicate that Dynamic-GLEP tends to capture receptor conformation-dependent interaction patterns associated with ligand efficacy. Although the present study did not include a systematic evaluation of structurally similar ligands with differing efficacy labels, the model’s consistent performance on chemically diverse external compounds suggests that its predictions are not solely driven by ligand scaffold similarity. Instead, the results imply that Dynamic-GLEP leverages features linked to receptor conformational states to make efficacy-relevant predictions. This observation highlights the framework’s potential to support the discovery of novel scaffolds while also underscoring the need for future targeted analyses to further validate these trends.

**Figure 8:**
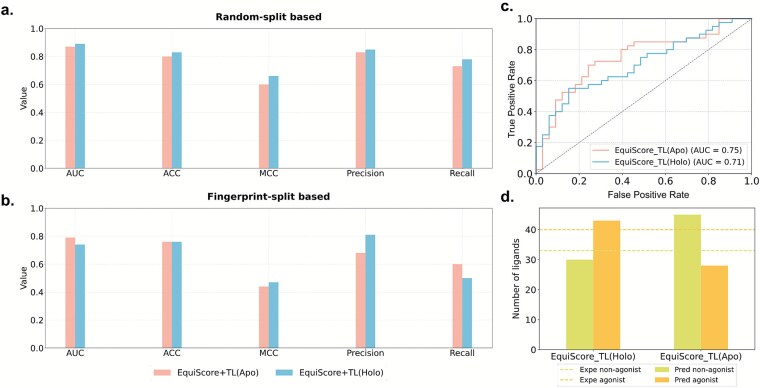
Performance of apo and holo structure modeling based on the 5-HT1A receptor. (a) Model performance on the random split strategy. (b) Model performance on the structural similarity–based split strategy. (c) ROC curves comparing EquiScore_TL (Apo) with EquiScore_TL (Holo). (d) Comparative statistics of predicted labels versus experimental labels.

### Holo versus Apo conformations: implications for G protein–coupled receptor ligand efficacy prediction

The initial receptor conformation plays a pivotal role in GPCR ligand efficacy prediction. Within a dynamic modeling framework, it defines the accessible conformational landscape during MD simulations and shapes the diversity and plausibility of protein–ligand binding modes, thereby influencing model accuracy and interpretability. Ligand-bound (Holo) structures are often favored due to their pre-stabilized and druggable binding pockets, whereas ligand-free (Apo) conformations remain essential for GPCRs without available Holo crystal structures.

To systematically evaluate the impact of starting conformations, we established two Dynamic-GLEP workflows for the 5-HT1A receptor: a Holo-based protocol (active: PDB 7E2Z; inactive: 5-HT2B-derived model from PDB 5 V54) and an Apo-based protocol (inactive: PDB 7E2X; active: 5-HT2B-derived model). Both pipelines were identical in MD simulation parameters, ligand datasets, conformational selection, and neural network architecture to ensure fair comparison.

Performance comparisons (Fig. 8a and b) revealed complementary characteristics. The Holo-based model achieved slightly higher accuracy under random-split validation, reflecting its advantage in recognizing known scaffolds and guiding structure–activity relationship (SAR) optimization. In contrast, the Apo-based model exhibited superior performance under structural similarity–based splits, indicating improved generalization toward novel scaffolds. The analysis confirmed that apo-protein simulations explored a broader conformational space, particularly in TM6 displacement, yet such flexibility may risk local pocket collapse, necessitating cautious application in virtual screening. External validation using IUPHAR ligands further supported this trend: Apo-based EquiScore_TL achieved higher overall AUC and accuracy, suggesting better robustness across chemically diverse ligands, whereas the Holo-based model showed higher precision for active compounds, benefiting from more structurally constrained binding sites.

In summary, Holo and Apo modeling strategies provide complementary advantages. The Holo-based workflow is better suited for lead optimization and SAR refinement, while the Apo-based approach, when combined with rational MD sampling and conformation filtering, facilitates chemical space exploration, especially for GPCRs lacking high-resolution ligand-bound structures. Explicitly assessing both conformational starting points is therefore essential to develop models that balance predictive accuracy, generalizability, and practical utility in GPCR-targeted drug discovery.

### Cross-target generalizability assessment using the A2A receptor

**Figure 9:**
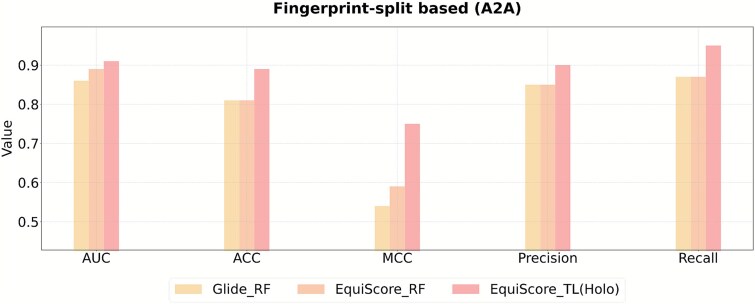
Model performance of modeling based on the A2A receptor holo structure under the structural similarity–based split strategy.

To evaluate Dynamic-GLEP’s cross-target generalizability, we selected the adenosine A2A receptor (A2A), which is structurally, chemically, and functionally distinct from 5-HT1A (e.g. TM3–TM6 motion, coupling pocket opening differences), to mimic data-scarce, conformationally variable scenarios (Fig. 9). Consistent with the 5-HT1A workflow, we built A2A active (PDB: 4UHR) and inactive (PDB: 3UZA) conformational ensembles via MD simulations, and curated a small representative dataset (75 ligands: 22 agonists, 53 nonagonists) from ChEMBL (smaller than 5-HT1A’s dataset, a key generalizability variable). The A2A-specific EquiScore_TL model showed high performance: AUC 0.89 ± 0.03 (random split) and 0.85 ± 0.02 (structural similarity split).

This confirms Dynamic-GLEP’s high accuracy despite variations in receptor conformations, data size, and ligands. Combined with 5-HT1A results, it shows that structural dynamics compensate for data scarcity, validating its use as a unified framework for GPCR drug discovery.

## Conclusion

In this work, we developed Dynamic-GLEP, a structure- and mechanism-aware deep learning framework that integrates GPCR conformational ensemble modeling with transfer learning based on equivariant graph neural networks for ligand efficacy prediction. By leveraging MD-derived representative conformations and integrating structural annotations into deep geometric representations, Dynamic-GLEP enables accurate classification of agonists versus nonagonists while improving model robustness and generalizability. Systematic evaluation on the 5-HT1A receptor demonstrated that Dynamic-GLEP achieved consistently high performance across both random and scaffold-based data splits and retained stable accuracy on an external FDA-related test set. Interpretability analysis revealed that the model captures conformation-dependent interaction patterns rather than relying on scaffold similarity, underscoring its mechanistic and structural generalizability. Comparative modeling further highlighted the complementary value of receptor starting states. Holo-based models provided superior performance for lead optimization and SAR refinement, whereas Apo-based models, with their broader conformational flexibility, better captured novel scaffolds and expanded the accessible chemical space, which is particularly valuable for GPCRs lacking high-resolution Holo structures. Extending the framework to the adenosine A₂A receptor further validated its cross-target transferability, maintaining strong predictive accuracy despite distinct activation mechanisms and limited training data.

While Dynamic-GLEP is substantially more efficient than free energy perturbation-based approaches, its multi-stage workflow still incurs notable computational cost. Future optimizations, such as coarse-grained or mixed-resolution simulations, adaptive-sampling strategies guided by model uncertainty, and lightweight or distilled neural architectures, could further improve scalability for large-scale virtual screening. Although this study focused on representative Class A GPCRs, the modular architecture of Dynamic-GLEP allows straightforward adaptation to Classes B and C, which feature extensive extracellular domains and multi-domain activation mechanisms. Incorporating extracellular-domain dynamics into ensemble modeling and redefining efficacy annotations to reflect these complex activation pathways will be key to broadening its applicability. These directions will be pursued in future work to generalize the framework across GPCR superfamilies. Collectively, Dynamic-GLEP establishes a reliable, interpretable, and extensible platform for GPCR ligand efficacy prediction. By bridging receptor dynamics with machine learning, it provides a practical foundation for mechanism-aware virtual screening, candidate prioritization, and rational GPCR drug design, advancing the integration of structural biology and artificial intelligence (AI)-driven cheminformatics.

**Key Points**

- Dynamic-GLEP integrates molecular dynamics–derived G protein–coupled receptor (GPCR) conformational ensembles with equivariant deep learning for ligand efficacy prediction.
- Ensemble docking combined with transfer learning enables recognition of conformation-dependent protein–ligand interaction patterns beyond static structures.
- The framework delivers robust agonist/nonagonist classification across representative Class A GPCRs with strong scaffold-level generalization.
- Holo- and apo-based modeling provide complementary strategies for GPCR virtual screening and lead optimization, including data-limited targets.

## Supplementary Material

A1-BIB-25-1837_SI_bbag049

## Data Availability

The data underlying this article, including the processed molecular docking scores and the penultimate layer features used for transfer learning, are available in the GitHub repository at https://github.com/orange2350/Dynamic-GLEP. The source code for model implementation is also provided in the same repository. The raw MD trajectory data are not publicly available due to their extremely large file size but will be shared on reasonable request to the corresponding author.
