# Supplementary material for: Dynamic-GLEP: a dynamics-informed deep learning framework for ligand efficacy prediction in representative Class A GPCRs
Source: Brief Bioinform. 2026 Feb 12;27(1):bbag049. doi: 10.1093/bib/bbag049 (PMC12900074; doi:10.1093/bib/bbag049)
Supplement: A1-BIB-25-1837_SI_bbag049 [file a1-bib-25-1837_si_bbag049.doc]

Supporting Information for

**Dynamic-GLEP: A Dynamics-Informed Deep Learning Framework for Ligand Efficacy Prediction in Representative Class A GPCRs**

Zhiyi Chen^1,2#^, Yongxin Hao^2,3#^, Yuhong Su^4#^, Hans Ågren^6^, Mingan Chen^2,7^, Zhehuan Fan^2,5^, Duanhua Cao^2,8^, Jiacheng Xiong^2,5^, Wei Zhang^2,5^, Jin Liu^2,8^, Xutong Li^2,5^, Mingyue Zheng^1,2^, Xi Cheng^2,5*^, Dingyan Wang^4*^, Dan Teng^2,5*^

*^1^School of Life Sciences, Nanjing University, Nanjing, 210023, China*

*^2^Drug Discovery and Design Center, State Key Laboratory of Drug Research, Shanghai Institute of Materia Medica, Chinese Academy of Sciences, 555 Zuchongzhi Road, Shanghai 201203, China*

*^3^Division of Life Science and Medicine, University of Science and Technology of China, Hefei, 230026, Anhui, China*

*^4^Lingang Laboratory, Shanghai 200031, China*

*^5^University of Chinese Academy of Sciences, No. 19A Yuquan Road, Beijing 100049, China*

*^6^Division of X-ray Photon Science, Department of Physics and Astronomy, Uppsala University, Box 516, SE-751 20Uppsala, Sweden*

*^7^School of Physical Science and Technology, ShanghaiTech University, Shanhai,201210, China*

*^8^Innovation Institute for Artificial Intelligence in Medicine of Zhejiang University, College of Pharmaceutical Sciences, Zhejiang University, Hangzhou, Zhejiang 310058, China*

#These authors Contribute equally

*Corresponding Author, Dan Teng, Dingyan Wang, Xi Cheng.

Email: tengdan@simm.ac.cn; wangdy@lglab.ac.cn; [xicheng@simm.ac.cn](mailto:xicheng@simm.ac.cn)

**SUPPLEMENTARY INFORMATION**

**CONTENTS:**

| **S.No.** | **Title** | **Page** |
| --- | --- | --- |
| 1 | **Figure S1.** Structural formulas and interpretability analysis diagrams of L-694,247 and Flurocarazolol**.** | 3 |
| 2 | **Table S1**: The distribution of labels in the C-5HT1A dataset following both random and fingerprint-based split, as well as the label distribution within the I-5HT1A dataset | 4 |
| 3 | **Table S2**: Processed C-5HT1A set | 4-19 |
| 4 | **Table S3**: Processed I-5HT1A set | 20-22 |

**
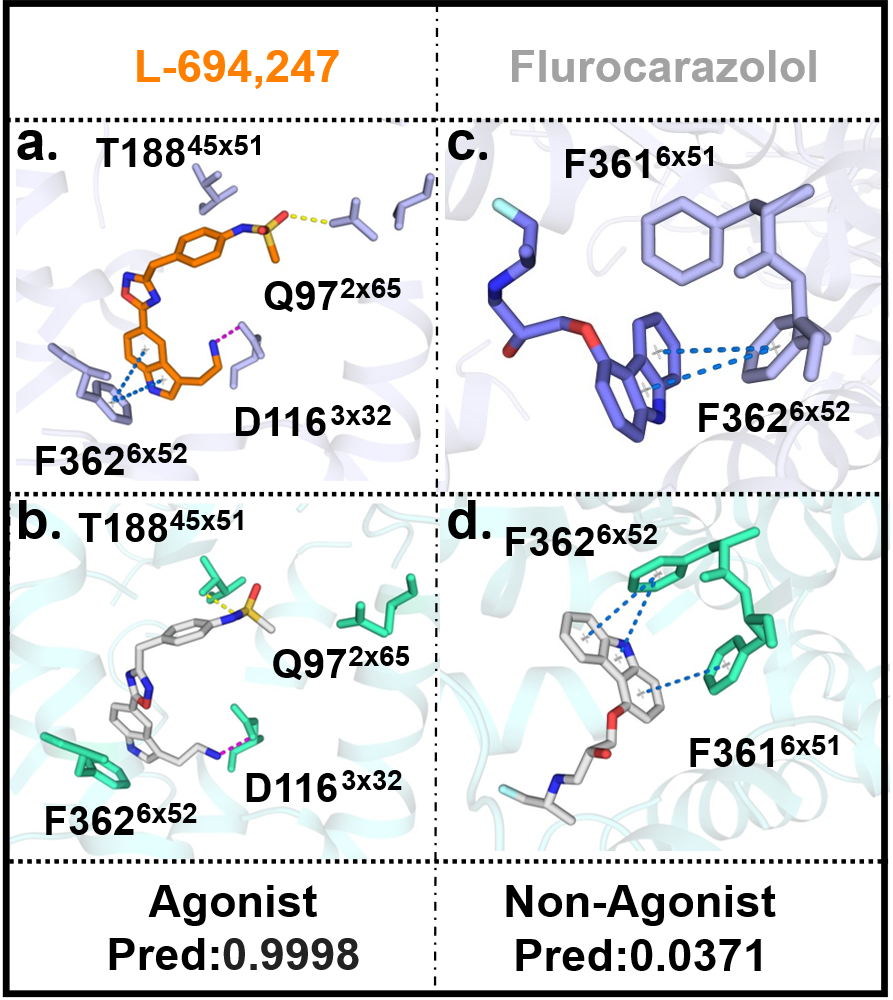
**

**Figure S1**. Structural formulas and interpretability analysis of L-694,247 and Flucolarazole. (a, b) In the active conformation (a), L-694,247 forms two π- π interactions with F362^6×52^, a salt bridge with D362^3×32^, and a hydrogen bond with Q97^2×65^. In the inactive conformation (b), it forms a salt bridge with D362^3×32^. (c, d) In the active conformation (c), Flucolarazole forms two π- π interactions with F362^6×52^. In the inactive conformation (d), it forms one π- π interaction with F361^6×51^ and two π- π interactions with F362^6×52^.

**Table S1**: The distribution of labels in the C-5HT1A dataset following both random and fingerprint-based split, as well as the label distribution within the I-5HT1A dataset

| **Dataset** | **Splitting Methods** |  | **Agonist** | **Antagonist** |
| --- | --- | --- | --- | --- |
| **C-5HT1A** | **Random** | **first fold** | **56** | **52** |
|  |  | **Second fold** | **59** | **49** |
|  |  | **Third fold** | **57** | **51** |
|  | **Fingerprint** | **First fold** | **58** | **50** |
|  |  | **Second fold** | **53** | **55** |
|  |  | **Third fold** | **36** | **72** |
| **I-5HT1A** | **-** | **-** | **55** | **39** |

**Table S2**: Processed C-5HT1A set

| ChEMBL ID | SMIES | Label | Emax_Label |
| --- | --- | --- | --- |
| CHEMBL8618 | COc1ccccc1N1CCN(CCCCN2C(=O)c3ccccc3C2=O)CC1 | 1.8 | 0 |
| CHEMBL608728 | C=CCOc1cccc2c1-c1cccc3c1[C@@H](C2)N(C)CC3 | 61 | 1 |
| CHEMBL60748 | Cc1ccc2cc(C3CCN(C[C@H](O)COc4cccc5[nH]ccc45)CC3)sc2c1 | 13.85 | 0 |
| CHEMBL606864 | N#Cc1ccc2[nH]cc(CCCNC3COc4ccc5c(c4C3)C(=O)NC5)c2c1 | 91 | 1 |
| CHEMBL606149 | O=C1NCCc2ccc3c(c21)CC(NCCCc1c[nH]c2ccc(F)cc12)CO3 | 87 | 1 |
| CHEMBL604513 | COc1cccc2c(CCN3CC=C(c4c[nH]c5cc(F)ccc45)CC3)coc12 | 100 | 1 |
| CHEMBL604063 | CN(CCCc1c[nH]c2ccc(F)cc12)C1COc2ccc3c(c2C1)C(=O)NCC3 | 99 | 1 |
| CHEMBL609317 | C[C@@H]1Cc2ccc3c(c2O1)-c1cccc2c1[C@@H](C3)N(C)CC2 | 127 | 1 |
| CHEMBL596885 | C#CCOc1cccc2c1-c1cccc3c1[C@@H](C2)N(C)CC3 | 73 | 1 |
| CHEMBL595744 | O=C1NCCc2cc(F)c3c(c21)CC(NCCCc1c[nH]c2ccc(F)cc12)CO3 | 98 | 1 |
| CHEMBL595622 | O=C1NCc2ccc3c(c21)CC(NCCCCc1c[nH]c2ccc(F)cc12)CO3 | 96 | 1 |
| CHEMBL595055 | CCN(CCCc1c[nH]c2ccc(F)cc12)C1COc2c(F)cc3c(c2C1)C(=O)NCC3 | 92 | 1 |
| CHEMBL594829 | O=C1NCc2ccc3c(c21)CC(NCCCc1c[nH]c2ccc(F)cc12)CO3 | 100 | 1 |
| CHEMBL594788 | CCN(CCCc1c[nH]c2ccc(F)cc12)C1COc2ccc3c(c2C1)C(=O)NCC3 | 97 | 1 |
| CHEMBL59437 | Cc1cc(C)c2cc(C3CCN(C[C@H](O)COc4cccc5[nH]c(C)cc45)CC3)sc2c1 | 13.38 | 0 |
| CHEMBL594357 | O=C1NCCc2ccc3c(c21)CC(N(CCCc1c[nH]c2ccc(F)cc12)C1CCC1)CO3 | 77 | 1 |
| CHEMBL593865 | O=C1NCCc2ccc3c(c21)CC(N(CCCc1c[nH]c2ccc(F)cc12)CC1CC1)CO3 | 91 | 1 |
| CHEMBL593281 | O=C1NCc2ccc3c(c21)CC(N(CCCc1c[nH]c2ccc(F)cc12)C(=O)c1ccccc1)CO3 | 96 | 1 |
| CHEMBL592953 | CCCN(CCCc1c[nH]c2ccc(F)cc12)C1COc2ccc3c(c2C1)C(=O)NCC3 | 100 | 1 |
| CHEMBL592951 | CC(C)N(CCCc1c[nH]c2ccc(F)cc12)C1COc2ccc3c(c2C1)C(=O)NCC3 | 94 | 1 |
| CHEMBL592939 | O=C1NCCc2cc(F)c3c(c21)CC(N(CCCc1c[nH]c2ccc(F)cc12)CC1CC1)CO3 | 62 | 1 |
| CHEMBL592029 | CCN(CCCc1c[nH]c2ccc(F)cc12)C1COc2ccc3c(c2C1)C(=O)NC3 | 78 | 1 |
| CHEMBL592028 | CN(CCCc1c[nH]c2ccc(F)cc12)C1COc2ccc3c(c2C1)C(=O)NC3 | 80 | 1 |
| CHEMBL589141 | O=C1NCc2ccc3c(c21)CC(N(CCCCc1c[nH]c2ccc(F)cc12)CC1CC1)CO3 | 87 | 1 |
| CHEMBL583752 | Fc1cccc(Oc2ccccc2C2CCNCC2)c1 | 51 | 1 |
| CHEMBL583751 | Fc1cccc(C2CCNCC2)c1Oc1ccccc1 | 35 | 0 |
| CHEMBL582945 | COc1ccccc1N1CCN(CCCn2cc(CCCCN3CCc4cc(O)c(O)cc4C(c4cccc(C)c4)C3)nn2)CC1 | 136 | 1 |
| CHEMBL575290 | Clc1cccc(Oc2ccccc2C2CCNCC2)c1 | 44 | 0 |
| CHEMBL574700 | COc1ccccc1N1CCN(CCCc2cn(CCCN3CCc4cc(O)c(O)cc4C(c4ccccc4C)C3)nn2)CC1 | 125 | 1 |
| CHEMBL574004 | Fc1cccc(Oc2cccc(F)c2C2CCNCC2)c1 | 74 | 1 |
| CHEMBL57385 | COc1cccc2cc(C3CCN(C[C@H](O)COc4cccc5[nH]ccc45)CC3)sc12 | 9.32 | 0 |
| CHEMBL57277 | COc1ccc2cc(C3CCN(C[C@H](O)COc4cccc5[nH]ccc45)CC3)sc2c1 | 4.92 | 0 |
| CHEMBL56795 | COc1cccc2sc(C3CCN(C[C@H](O)COc4cccc5[nH]ccc45)CC3)cc12 | 7.47 | 0 |
| CHEMBL56601 | O[C@H](COc1cccc2[nH]ccc12)CN1CCC(c2cc3ccc(F)cc3s2)CC1 | 11.65 | 0 |
| CHEMBL56303 | O[C@H](COc1cccc2[nH]ccc12)CN1CCC(c2cc3cc(Cl)ccc3s2)CC1 | 10.97 | 0 |
| CHEMBL56156 | Cc1cccc2sc(C3CCN(C[C@H](O)COc4cccc5[nH]ccc45)CC3)cc12 | 16.99 | 0 |
| CHEMBL56147 | O[C@H](COc1cccc2[nH]ccc12)CN1CCC(c2cc3cc(F)ccc3s2)CC1 | 13.23 | 0 |
| CHEMBL537403 | COc1ccccc1N1CCN(CCN(C(=O)C2CCCCC2)c2ccccn2)CC1 | 0 | 0 |
| CHEMBL522671 | Fc1ccc2c(c1)[nH]cc2[C@H]1CC[C@@H](N2CCN(c3cccc4[nH]ccc34)CC2)CC1 | 0 | 0 |
| CHEMBL521665 | COc1ccccc1OCCNC[C@@H]1CO[C@H](c2ccccc2)CO1 | 85.5 | 1 |
| CHEMBL521510 | COc1cccc2c(CCCN(C3CCC3)C3COc4c(F)ccc(C(N)=O)c4C3)c[nH]c12 | 0 | 0 |
| CHEMBL521341 | CN(C)C(=O)c1ccc(F)c2c1CC(N(CCCc1c[nH]c3ccc(F)cc13)C1CCC1)CO2 | 0 | 0 |
| CHEMBL521303 | O=C(O)c1ccc(F)c2c1CC(N(CCCc1c[nH]c3ccc(F)cc13)C1CCC1)CO2 | 0 | 0 |
| CHEMBL520994 | CCCN(CCCCn1ccc2ccc(F)cc21)C1COc2c(F)ccc(C(N)=O)c2C1 | 40 | 0 |
| CHEMBL520661 | NC(=O)c1ccc(F)c2c1CC(N(CCCc1c[nH]c3cc(F)ccc13)C1CCC1)CO2 | 0 | 0 |
| CHEMBL519679 | O=C(NC1CCC1)c1ccc(F)c2c1CC(N(CCCc1c[nH]c3ccc(F)cc13)C1CCC1)CO2 | 0 | 0 |
| CHEMBL519514 | NC(=O)c1ccc(F)c2c1CC(N(CCCCn1ccc3ccc(F)cc31)CC1CC1)CO2 | 0 | 0 |
| CHEMBL519325 | NC(=O)c1ccc(F)c2c1CC(N(CCCCn1ccc3cccc(F)c31)C1CCC1)CO2 | 0 | 0 |
| CHEMBL481337 | c1cc(N2CCN([C@H]3CC[C@@H](c4c[nH]c5ccccc54)CC3)CC2)c2nc[nH]c2c1 | 0 | 0 |
| CHEMBL516158 | CCCN(CCCc1c[nH]c2ccc(F)cc12)[C@H]1COc2cccc(C(N)=O)c2C1 | 74 | 1 |
| CHEMBL484210 | CCCN(CCCc1c[nH]c2ccc(F)cc12)[C@@H]1COc2c(F)ccc(OC)c2C1 | 94 | 1 |
| CHEMBL494483 | N#Cc1ccc2[nH]cc([C@H]3CC[C@@H](N4CCN(c5cccc6[nH]ccc56)CC4)CC3)c2c1 | 0 | 0 |
| CHEMBL2312225 | COc1cccc(OC)c1OCCNC[C@@H]1COCC(c2ccccc2)(c2ccccc2)O1 | 119.5 | 1 |
| CHEMBL2312227 | COc1ccccc1OCCNC[C@@H]1COCC(c2ccccc2)(c2ccccc2)O1 | 66.6 | 1 |
| CHEMBL493285 | c1ccc(OCCNC[C@@H]2COCC(c3ccccc3)(c3ccccc3)O2)cc1 | 72.7 | 1 |
| CHEMBL493037 | c1cc(N2CCN([C@H]3CC[C@@H](c4c[nH]c5ccccc54)CC3)CC2)c2cc[nH]c2c1 | 0 | 0 |
| CHEMBL4875081 | COc1ccc(Cl)cc1[C@H]1C[C@@H]1CNCCCOc1ccc2ccc(=O)[nH]c2c1 | 45 | 0 |
| CHEMBL4863832 | COc1ccc(F)cc1[C@H]1C[C@@H]1CNCCCOc1ccc2ccc(=O)[nH]c2c1 | 49 | 0 |
| CHEMBL4862890 | COc1ccc(F)cc1[C@H]1C[C@@H]1CNCCCCOc1ccc2ccc(=O)[nH]c2c1 | 58 | 1 |
| CHEMBL4862770 | COc1ccc(Cl)cc1[C@H]1C[C@@H]1CNCCCCOc1ccc2c(c1)NC(=O)CC2 | 38 | 0 |
| CHEMBL485625 | NC(=O)c1ccc(F)c2c1CC(N(CCCc1c[nH]c3c(F)cc(F)cc13)C1CCC1)CO2 | 0 | 0 |
| CHEMBL485624 | NC(=O)c1ccc(F)c2c1CC(N(CCCc1c[nH]c3c(Cl)cccc13)C1CCC1)CO2 | 0 | 0 |
| CHEMBL485579 | CCCN(CC1CCc2[nH]c3ccc(F)cc3c2C1)[C@H]1COc2c(F)ccc(C(N)=O)c2C1 | 0 | 0 |
| CHEMBL485434 | CNC(=O)c1ccc(F)c2c1C[C@@H](N(CCCc1c[nH]c3ccc(F)cc13)C1CCC1)CO2 | 0 | 0 |
| CHEMBL485407 | NC(=O)c1ccc(F)c2c1C[C@@H](NCC1CCc3[nH]c4ccc(F)cc4c3C1)CO2 | 0 | 0 |
| CHEMBL485398 | NC(=O)c1ccc(F)c2c1CC(N(CCCCn1ccc3ccc(F)cc31)C1CCC1)CO2 | 0 | 0 |
| CHEMBL485397 | NC(=O)c1ccc(F)c2c1CC(N(CCCCn1ccc3cc(F)ccc31)C1CCC1)CO2 | 0 | 0 |
| CHEMBL485385 | O=C(NC1CC1)c1ccc(F)c2c1CC(N(CCCc1c[nH]c3ccc(F)cc13)C1CCC1)CO2 | 0 | 0 |
| CHEMBL485381 | NC(=O)c1ccc(F)c2c1CC(N(CCCCn1ccc3c(F)cccc31)CC1CC1)CO2 | 0 | 0 |
| CHEMBL485228 | N#Cc1ccc2[nH]cc(CCCN(C3CCC3)C3COc4c(F)ccc(C(N)=O)c4C3)c2c1 | 0 | 0 |
| CHEMBL484948 | CC(C)NC(=O)c1ccc(F)c2c1CC(N(CCCc1c[nH]c3ccc(F)cc13)C1CCC1)CO2 | 0 | 0 |
| CHEMBL484947 | CCCNC(=O)c1ccc(F)c2c1CC(N(CCCc1c[nH]c3ccc(F)cc13)C1CCC1)CO2 | 0 | 0 |
| CHEMBL484581 | COc1ccc2[nH]cc(CCCN(C3CCC3)C3COc4c(F)ccc(C(N)=O)c4C3)c2c1 | 84 | 1 |
| CHEMBL484572 | NC(=O)c1ccc(Cl)c2c1CC(N(CCCc1c[nH]c3ccc(F)cc13)C1CCC1)CO2 | 0 | 0 |
| CHEMBL484541 | O=C(NCC1CC1)c1ccc(F)c2c1CC(N(CCCc1c[nH]c3ccc(F)cc13)C1CCC1)CO2 | 0 | 0 |
| CHEMBL484540 | O=C(NC1CCCCC1)c1ccc(F)c2c1CC(N(CCCc1c[nH]c3ccc(F)cc13)C1CCC1)CO2 | 0 | 0 |
| CHEMBL484537 | NC(=O)c1ccc(F)c2c1CC(N(CCCCn1ccc3cccc(F)c31)CC1CC1)CO2 | 31 | 0 |
| CHEMBL484355 | CCCN(CCCCn1ccc2cccc(F)c21)C1COc2c(F)ccc(C(N)=O)c2C1 | 39 | 0 |
| CHEMBL483594 | CCNC(=O)c1ccc(F)c2c1CC(N(CCCc1c[nH]c3ccc(F)cc13)C1CCC1)CO2 | 0 | 0 |
| CHEMBL483573 | NC(=O)c1ccc(F)c2c1CC(N(CCCc1c[nH]c3ccc(Cl)cc13)C1CCC1)CO2 | 0 | 0 |
| CHEMBL481754 | N#Cc1ccc2[nH]cc([C@H]3CC[C@@H](N4CCN(c5cccc6cccnc56)CC4)CC3)c2c1 | 23 | 0 |
| CHEMBL481747 | Fc1ccc2[nH]cc([C@H]3CC[C@H](N4CCN(c5cccc6cccnc56)CC4)CC3)c2c1 | 66 | 1 |
| CHEMBL520265 | FC(F)(F)c1nc2c(N3CCN([C@H]4CC[C@@H](c5c[nH]c6ccccc65)CC4)CC3)cccc2[nH]1 | 0 | 0 |
| CHEMBL519616 | Cc1nc2c(N3CCN([C@H]4CC[C@@H](c5c[nH]c6ccccc65)CC4)CC3)cccc2[nH]1 | 0 | 0 |
| CHEMBL518946 | N#Cc1ccc2[nH]cc([C@H]3CC[C@@H](N4CCN(c5cccc6nccnc56)CC4)CC3)c2c1 | 0 | 0 |
| CHEMBL481108 | Fc1ccc2[nH]cc([C@H]3CC[C@@H](N4CCN(c5cccc6ncccc56)CC4)CC3)c2c1 | 0 | 0 |
| CHEMBL520753 | N#Cc1ccc2[nH]cc([C@H]3CC[C@@H](N4CCN(c5cccc6ncccc56)CC4)CC3)c2c1 | 0 | 0 |
| CHEMBL479986 | Fc1ccc2[nH]cc([C@H]3CC[C@H](N4CCN(c5cccc6ccccc56)CC4)CC3)c2c1 | 71 | 1 |
| CHEMBL4797282 | O=C(c1ccc(F)c(Cl)c1)N1CCC(F)(CNCCOc2cccc(Cl)c2)CC1 | 91 | 1 |
| CHEMBL4796345 | CNc1cccc(OCCNCC2(F)CCN(C(=O)c3ccc(F)c(Cl)c3)CC2)c1 | 91 | 1 |
| CHEMBL4793515 | O=C(c1ccc(F)c(Cl)c1)N1CCC(F)(CNCCSc2ccccc2)CC1 | 75.33333 | 1 |
| CHEMBL4790662 | COc1cccc(OCCNCC2(F)CCN(C(=O)c3ccc(Cl)c(Cl)c3)CC2)c1 | 90 | 1 |
| CHEMBL4790600 | CC1(C)Cc2cccc(OCCNCC3(F)CCN(C(=O)c4ccc(F)c(Cl)c4)CC3)c2O1 | 89.25 | 1 |
| CHEMBL4786537 | O=C(c1ccc(F)c(Cl)c1)N1CCC(F)(CNCCCc2ccccn2)CC1 | 89.25 | 1 |
| CHEMBL4777581 | NC(=O)c1cccc(OCCNCC2(F)CCN(C(=O)c3ccc(F)c(Cl)c3)CC2)c1 | 96.25 | 1 |
| CHEMBL4760666 | CN(C)c1cccc(OCCNCC2(F)CCN(C(=O)c3ccc(F)c(Cl)c3)CC2)c1 | 90.25 | 1 |
| CHEMBL4760271 | O=C(c1ccc(F)c(Cl)c1)N1CCC(F)(CNCCOc2ccccc2F)CC1 | 92.75 | 1 |
| CHEMBL4759629 | O=C(c1ccc(F)c(Cl)c1)N1CCC(F)(CNCCSc2ccccn2)CC1 | 83.75 | 1 |
| CHEMBL4756233 | O=C(c1ccc(F)c(Cl)c1)N1CCC(F)(CNCCOc2cccc3[nH]ccc23)CC1 | 92.75 | 1 |
| CHEMBL4755058 | CC(=O)Nc1cccc(OCCNCC2(F)CCN(C(=O)c3ccc(F)c(Cl)c3)CC2)c1 | 96 | 1 |
| CHEMBL4754363 | O=C(c1ccc(F)c(Cl)c1)N1CCC(F)(CNCCCc2ccccc2)CC1 | 85.25 | 1 |
| CHEMBL4753414 | O=C(c1ccc(F)c(Cl)c1)N1CCC(F)(CNCCOc2cnccn2)CC1 | 94 | 1 |
| CHEMBL4752833 | O=C(c1ccc(Cl)c(Cl)c1)N1CCC(F)(CNCCOc2cccc(F)c2)CC1 | 94 | 1 |
| CHEMBL4752177 | CC(=O)Nc1cccc(OCCNCC2(F)CCN(C(=O)c3ccc(Cl)c(Cl)c3)CC2)c1 | 97.5 | 1 |
| CHEMBL4750444 | NC(=O)c1ccccc1OCCNCC1(F)CCN(C(=O)c2ccc(F)c(Cl)c2)CC1 | 93 | 1 |
| CHEMBL4747425 | O=C(c1ccc(F)c(Cl)c1)N1CCC(F)(CNCCOc2ccc(F)cc2)CC1 | 91.5 | 1 |
| CHEMBL4742122 | O=C(c1ccc(F)c(Cl)c1)N1CCC(F)(CNCCOc2cccnc2)CC1 | 99.5 | 1 |
| CHEMBL4739908 | O=C(c1ccc(F)c(Cl)c1)N1CCC(F)(CNCCOc2cccc(F)c2)CC1 | 89.75 | 1 |
| CHEMBL4591601 | COc1ccc(/C=C2\NC(=O)N(CCCCN3CCN(c4cccc(Cl)c4Cl)CC3)C2=O)cc1OC | 13.5 | 0 |
| CHEMBL4588223 | COc1ccccc1OCCNCC1COC(c2ccccc2)(c2ccccc2)OC1 | 91.6 | 1 |
| CHEMBL4582160 | c1ccc(OCCNCC2CCOC(c3ccccc3)(c3ccccc3)O2)cc1 | 79.5 | 1 |
| CHEMBL4551941 | COc1ccccc1OCCNCC[C@@H]1CO[C@H](C(c2ccccc2)c2ccccc2)O1 | 82.1 | 1 |
| CHEMBL457750 | CCCN(CCCc1c[nH]c2ccc(F)cc12)[C@H]1COc2c(F)ccc(C(=O)NC)c2C1 | 0 | 0 |
| CHEMBL456657 | NC(=O)c1ccc(F)c2c1C[C@@H](N(CC1CC1)CC1CCc3[nH]c4ccc(F)cc4c3C1)CO2 | 0 | 0 |
| CHEMBL4558985 | OCC(CNCCOc1ccccc1)OC(c1ccccc1)c1ccccc1 | 69.5 | 1 |
| CHEMBL4554558 | C1=C(c2c[nH]c3ccccc23)CCN(Cc2cccs2)C1 | 67 | 1 |
| CHEMBL4552542 | COc1cccc(CN2CC=C(c3c[nH]c4ccccc34)CC2)c1 | 76 | 1 |
| CHEMBL4544457 | c1ccc(OCCNCC2COC(c3ccccc3)(c3ccccc3)OC2)cc1 | 88.7 | 1 |
| CHEMBL45305 | Cc1ccc(CNCC2(F)CCN(C(=O)c3ccc(F)c(Cl)c3)CC2)nc1 | 74.7 | 1 |
| CHEMBL452437 | COc1ccccc1OCCNC[C@@H]1COC[C@H](c2ccccc2)O1 | 78.1 | 1 |
| CHEMBL448891 | COc1cc2c(cc1OC)[C@@H]1Cc3ccc(O)c(OC)c3CN1CC2 | 156.4 | 1 |
| CHEMBL4468507 | COc1ccccc1OCCNCCOC(c1ccccc1)c1ccccc1 | 77.3 | 1 |
| CHEMBL4467585 | COc1ccccc1OCCNCC1CCOC(c2ccccc2)(c2ccccc2)O1 | 65.9 | 1 |
| CHEMBL4466677 | COc1ccccc1N1CCN(CC2COCC(c3ccccc3)(c3ccccc3)O2)CC1 | 39.3 | 0 |
| CHEMBL4465959 | COc1ccc(/C=C2\NC(=O)N(CCCCN3CCN(c4ccccc4OC)CC3)C2=O)cc1OC1CCCC1 | 4.5 | 0 |
| CHEMBL4465749 | COc1ccccc1OCCNC[C@H]1OCCO[C@H]1c1ccccc1 | 24.3 | 0 |
| CHEMBL4462600 | c1ccc(OCCNCCOC(c2ccccc2)c2ccccc2)cc1 | 78.9 | 1 |
| CHEMBL4458714 | COc1ccccc1OCCNCCC1COC(c2ccccc2)(c2ccccc2)O1 | 22.1 | 0 |
| CHEMBL4444133 | c1ccc(OCCNCC2OCCOC2(c2ccccc2)c2ccccc2)cc1 | 32.9 | 0 |
| CHEMBL4439658 | COc1ccccc1OCCNCC(CO)OC(c1ccccc1)c1ccccc1 | 90.2 | 1 |
| CHEMBL441546 | C1=C(c2cccc(CNCCOc3cccc4c3OCO4)c2)CCC1 | 70.7 | 1 |
| CHEMBL441316 | O[C@H](COc1cccc2[nH]ccc12)CN1C2C=C(c3ccc(Cl)c(Cl)c3)CC1CC2 | 0 | 0 |
| CHEMBL440525 | Cc1cc2c(OC[C@@H](O)CN3CC[C@@H](c4cc5cc(Cl)ccc5s4)C[C@@H]3C)cccc2[nH]1 | 8.42 | 0 |
| CHEMBL438619 | CN1CCc2c(sc3ncn(CCN4CCN(c5cccc6ccncc56)CC4)c(=O)c23)C1 | 10 | 0 |
| CHEMBL435279 | COc1cccc2c1O[C@@H](CN1[C@@H]3CC[C@H]1C[C@](O)(c1cccc(C(F)(F)F)c1)C3)CO2 | 90 | 1 |
| CHEMBL435111 | C[C@H](CN(C(=O)c1ccc(C(F)(F)F)cc1)c1ccccn1)N1CCN(c2cccc3c2OCCO3)CC1 | 0 | 0 |
| CHEMBL434440 | Cc1cc2c(OC[C@@H](O)CN3CC[C@@H](c4cc5ccc(F)cc5s4)C[C@@H]3C)cccc2[nH]1 | 7.26 | 0 |
| CHEMBL426317 | COc1cccc(N2CCN(CCCCn3ncc(=O)n(C)c3=O)CC2)c1 | 95 | 1 |
| CHEMBL425936 | COc1ccc2c(c1)OC(CNCCc1c[nH]c3ccc(F)cc13)CN2 | 56 | 1 |
| CHEMBL425833 | O=C(c1ccc(F)c(Cl)c1)N1CCC(F)(CNCc2ccc(Cl)cn2)CC1 | 80.4 | 1 |
| CHEMBL421950 | COc1ccccc1[C@]1(O)C[C@H]2CC[C@@H](C1)N2C[C@H]1COc2cccc(OC)c2O1 | 40 | 0 |
| CHEMBL421660 | Cc1cc2c(OC[C@@H](O)CN3CC[C@@H](c4cc5ccccc5s4)C[C@@H]3C)cccc2[nH]1 | 6.045 | 0 |
| CHEMBL4213352 | COc1ccccc1N1CCN(CCOCCOc2cc(C)cc(C)c2C)CC1 | 4.5 | 0 |
| CHEMBL421328 | COc1cccc2sc([C@@H]3C[C@H]4CC[C@@H](C3)N4C[C@H](O)COc3cccc4[nH]ccc34)cc12 | 7.22 | 0 |
| CHEMBL4212812 | Cc1cc(OCCCN2CCN(c3ccccc3)CC2)ccc1Cl | 8.5 | 0 |
| CHEMBL4209888 | COc1ccccc1N1CCN(CCCOc2ccc(C)cc2C)CC1 | 3.5 | 0 |
| CHEMBL4207653 | COc1ccccc1N1CCN(CCOCCOc2c(C)cc(C)cc2C)CC1 | 11.5 | 0 |
| CHEMBL4205526 | COc1ccc(N2CCN(CCCOc3c(C)cc(C)cc3C)CC2)cc1 | 4.5 | 0 |
| CHEMBL417782 | Cc1cc2c(OC[C@@H](O)CN3CCC(c4cc5c(F)cccc5s4)CC3)cccc2[nH]1 | 11.62 | 0 |
| CHEMBL413777 | CC(C)Oc1ccccc1OCCNCc1cccc(C2CCCC2)c1 | 71.2 | 1 |
| CHEMBL4130132 | CC(=O)c1c(OCCCCN2CCN(c3ccccc3C#N)CC2)ccc2c(C)cc(=O)oc12 | 2 | 0 |
| CHEMBL4129562 | CC(=O)c1c(OCCCN2CCN(c3ccccc3F)CC2)ccc2c(C)cc(=O)oc12 | 2.5 | 0 |
| CHEMBL4129293 | CC(=O)c1c(OCCCN2CCN(c3ccccc3C#N)CC2)ccc2c(C)cc(=O)oc12 | 1.5 | 0 |
| CHEMBL4129173 | COc1cccc(N2CCN(CCCCOc3ccc4c(C)cc(=O)oc4c3C(C)=O)CC2)c1 | 1.5 | 0 |
| CHEMBL4128053 | COc1cccc(N2CCN(CCCOc3ccc4c(C)cc(=O)oc4c3C(C)=O)CC2)c1 | 1.5 | 0 |
| CHEMBL4127564 | CC(=O)c1c(OCCCCN2CCN(c3ccccc3F)CC2)ccc2c(C)cc(=O)oc12 | 1.5 | 0 |
| CHEMBL4126092 | CC(=O)c1c(OCCCCN2CCN(c3cccc(Cl)c3Cl)CC2)ccc2c(C)cc(=O)oc12 | 1.5 | 0 |
| CHEMBL410273 | Fc1ccc2[nH]cc(CCNCC3CNc4ccccc4O3)c2c1 | 100 | 1 |
| CHEMBL4091168 | c1ccc(C2(c3ccccc3)COCC(CNCCOc3ccccn3)O2)cc1 | 63.9 | 1 |
| CHEMBL4082195 | Oc1ccccc1OCCNCC1COCC(c2ccccc2)(c2ccccc2)O1 | 65.6 | 1 |
| CHEMBL4075507 | Clc1ccccc1OCCNCC1COCC(c2ccccc2)(c2ccccc2)O1 | 66 | 1 |
| CHEMBL4071929 | COCOc1ccccc1OCCNCC1COCC(c2ccccc2)(c2ccccc2)O1 | 62.8 | 1 |
| CHEMBL4070363 | Cc1ccccc1OCCNCC1COCC(c2ccccc2)(c2ccccc2)O1 | 59.7 | 1 |
| CHEMBL4067083 | O=[N+]([O-])c1ccccc1OCCNCC1COCC(c2ccccc2)(c2ccccc2)O1 | 82.5 | 1 |
| CHEMBL396062 | CNc1nc(CNCC2(F)CCN(C(=O)c3ccc(F)c(Cl)c3)CC2)ncc1Cl | 71.8 | 1 |
| CHEMBL394778 | CN(C)c1ccnc(CNCC2(F)CCN(C(=O)c3ccc(F)c(Cl)c3)CC2)n1 | 63.4 | 1 |
| CHEMBL394606 | CNc1nc(CNCC2(F)CCN(C(=O)c3ccc(F)c(Cl)c3)CC2)ncc1C | 64.1 | 1 |
| CHEMBL394530 | O=C(c1ccc(F)c(Cl)c1)N1CCC(F)(CNCc2ccc(CF)cn2)CC1 | 61 | 1 |
| CHEMBL3938383 | COc1ccccc1OCCNCC1CSC2(CCCCC2)S1 | 74 | 1 |
| CHEMBL3929328 | COc1ccccc1N1CCN(CC2CSC3(CCCCC3)S2)CC1 | 37 | 0 |
| CHEMBL3917316 | c1ccc(OCCNCC2CSC3(CCCCC3)S2)cc1 | 85 | 1 |
| CHEMBL391531 | Fc1ccc2[nH]cc(CCCNC3COc4c(ccc5ncccc45)C3)c2c1 | 87.5 | 1 |
| CHEMBL391230 | OC(CCNC12CC3CC(CC(C3)C1)C2)COc1cccc2[nH]ccc12 | 51.8 | 1 |
| CHEMBL386988 | CC(C)Oc1ccccc1OCCNCc1cccc(-c2ccsc2)c1 | 47.7 | 0 |
| CHEMBL385353 | NC(=O)c1ccc(F)c2c1CC(N(CCCCc1c[nH]c3ccc(F)cc13)CC1CC1)CO2 | 0 | 0 |
| CHEMBL384657 | CC1(C)Cc2cccc(OCCNCc3cncc(C4=CCCC4)c3)c2O1 | 72.4 | 1 |
| CHEMBL383688 | COc1cccc2c1OC(CN1CC=C(c3c[nH]c4ccc(F)cc34)CC1)CN2 | 35 | 0 |
| CHEMBL383446 | CC(C)N1CC(CNCCc2c[nH]c3ccc(F)cc23)Oc2ccccc21 | 90 | 1 |
| CHEMBL38288 | COc1ccccc1N1CCN(C[C@@H](C(=O)NC(C)(C)C)c2ccccc2)CC1 | 0.5 | 0 |
| CHEMBL382857 | CN1CC(CNCCc2c[nH]c3ccc(F)cc23)Oc2cc(Cl)ccc21 | 64 | 1 |
| CHEMBL382333 | CCCN1CC(CN2CC=C(c3c[nH]c4ccc(F)cc34)CC2)Oc2c(OC)cccc21 | 92 | 1 |
| CHEMBL382179 | CCCN1CC(CNCCc2c[nH]c3ccccc23)Oc2ccccc21 | 100 | 1 |
| CHEMBL381542 | c1ccc(CN2CC(CNCCCc3c[nH]c4ccccc34)Oc3ccccc32)cc1 | 64 | 1 |
| CHEMBL380401 | NC(=O)c1ccc(F)c2c1C[C@@H](NCCCc1c[nH]c3ccc(F)cc13)CO2 | 85 | 1 |
| CHEMBL380342 | c1ccc2c(c1)NCC(CNCCc1c[nH]c3ccccc13)O2 | 96 | 1 |
| CHEMBL379298 | NC(=O)c1ccc(F)c2c1CC(N(CCc1c[nH]c3ccc(F)cc13)CC1CC1)CO2 | 0 | 0 |
| CHEMBL378073 | CCCN(CCCc1c[nH]c2ccc(F)cc12)C1COc2cccc(OC)c2C1 | 90 | 1 |
| CHEMBL377941 | CN1CC(CNCCc2c[nH]c3ccc(F)cc23)Oc2ccccc21 | 80 | 1 |
| CHEMBL375297 | C1=C(c2cccc(CNCCOc3cccc4c3OCCO4)c2)CCC1 | 68.9 | 1 |
| CHEMBL3739897 | c1ccc(OCCNC[C@@H]2COC(c3ccccc3)(c3ccccc3)O2)cc1 | 65.3 | 1 |
| CHEMBL192020 | C[C@H](CN(C(=O)c1ccc(C#N)cc1)c1ccccn1)N1CCN(c2cccc3c2OCCO3)CC1 | 0 | 0 |
| CHEMBL371221 | CN1CCc2c(sc3ncn(CCN4CCN(c5nccc6ccccc56)CC4)c(=O)c23)C1 | 10 | 0 |
| CHEMBL192092 | C[C@H](CN(C(=O)c1ccc(Cl)cc1)c1ccccn1)N1CCN(c2cccc3c2OCCO3)CC1 | 0 | 0 |
| CHEMBL371094 | CN1CCc2c(sc3ncn(CCN4CCN(c5ccccc5OCC(C)(C)C)CC4)c(=O)c23)C1 | 10 | 0 |
| CHEMBL370110 | CN1CCc2c(sc3ncn(CCN4CCN(c5cncc6ccccc56)CC4)c(=O)c23)C1 | 4 | 0 |
| CHEMBL366091 | C1=C(c2cc3ccccc3[nH]2)CC2CCC1N2CCOc1cccc2ncccc12 | 0 | 0 |
| CHEMBL363700 | Cn1ccc2c(OCCN3C4C=C(c5ccc6ccccc6c5)CC3CC4)cccc21 | 100 | 1 |
| CHEMBL362430 | Fc1ccc2[nH]cc([C@H]3CC[C@H](NCC4COc5ccccc5O4)CC3)c2c1 | 94 | 1 |
| CHEMBL361916 | C1=C(c2c[nH]c3ccccc23)CC2CCC1N2CCCOc1cccc2[nH]ccc12 | 14 | 0 |
| CHEMBL361526 | Fc1ccc2[nH]cc([C@H]3CC[C@H](N4CCN(c5cccc6c5OCCO6)CC4)CC3)c2c1 | 57 | 1 |
| CHEMBL360637 | C1=C(c2c[nH]c3ccccc23)CC2CCC1N2CCOc1cccc2[nH]ccc12 | 0 | 0 |
| CHEMBL178467 | Fc1ccc2[nH]cc([C@H]3CC[C@H](NCCOc4cccc5[nH]ccc45)CC3)c2c1 | 59 | 1 |
| CHEMBL180817 | COc1ccccc1OCCN[C@H]1CC[C@H](c2c[nH]c3ccc(F)cc32)CC1 | 80 | 1 |
| CHEMBL359495 | Fc1ccc2[nH]cc([C@H]3CC[C@@H](N4CCN(c5cccc6[nH]ccc56)CC4)CC3)c2c1 | 10 | 0 |
| CHEMBL344432 | COc1cccc2c1CCCC2CCCNCCOc1ccccn1 | 86.8 | 1 |
| CHEMBL339086 | COc1ccc2cc([C@@H]3CCN(C[C@H](O)COc4cccc5[nH]c(C)cc45)[C@@H](C)C3)sc2c1 | 3.21 | 0 |
| CHEMBL338632 | Cc1cc2c(OC[C@@H](O)CN3CC[C@@H](c4cc5cccc(F)c5s4)C[C@@H]3C)cccc2[nH]1 | 4.09 | 0 |
| CHEMBL3342875 | CCCCNC(=O)N1CCC2(CC1)OCC(CN1CCN(c3ccccc3OC)CC1)O2 | 30 | 0 |
| CHEMBL3342874 | COc1ccccc1N1CCN(CC2COC3(CCN(C(=O)Nc4ccccc4)CC3)O2)CC1 | 28 | 0 |
| CHEMBL3342870 | COc1ccccc1N1CCN(CC2COC3(CCN(S(=O)(=O)c4ccc([N+](=O)[O-])cc4)CC3)O2)CC1 | 37 | 0 |
| CHEMBL3342867 | COc1ccccc1N1CCN(CC2COC3(CCN(S(=O)(=O)c4ccc(C)cc4)CC3)O2)CC1 | 28 | 0 |
| CHEMBL3342862 | COc1ccccc1N1CCN(CC2COC3(CCN(C(=O)C4CCCCC4)CC3)O2)CC1 | 33 | 0 |
| CHEMBL3342861 | COc1ccccc1N1CCN(CC2COC3(CCN(C(=O)c4ccco4)CC3)O2)CC1 | 33 | 0 |
| CHEMBL3342858 | COc1ccccc1N1CCN(CC2COC3(CCN(c4ccccn4)CC3)O2)CC1 | 41 | 0 |
| CHEMBL3342857 | COc1ccccc1N1CCN(CC2COC3(CCN(c4ccccc4)CC3)O2)CC1 | 42 | 0 |
| CHEMBL3342854 | COc1ccccc1N1CCN(CC2COC3(CC[S+](O)CC3)O2)CC1 | 139 | 1 |
| CHEMBL3342853 | COc1ccccc1N1CCN(CC2COC3(CCN(Cc4ccccc4)CC3)O2)CC1 | 45 | 0 |
| CHEMBL3342852 | COc1ccccc1N1CCN(CC2COC3(CCSCC3)O2)CC1 | 36 | 0 |
| CHEMBL3341769 | COc1ccccc1N1CCN(CC2COC3(CCOCC3)O2)CC1 | 31 | 0 |
| CHEMBL3330616 | Cn1c(=O)cnn(CCCCN2CCN(c3cccc(F)n3)CC2)c1=O | 95 | 1 |
| CHEMBL3330614 | Cn1c(=O)cnn(CCCCN2CCN(c3ncccc3F)CC2)c1=O | 80 | 1 |
| CHEMBL3330612 | Cn1c(=O)cnn(CCCCN2CCN(c3ccccn3)CC2)c1=O | 85 | 1 |
| CHEMBL3330603 | Cn1c(=O)cnn(CCCCN2CCN(c3ccccc3)CC2)c1=O | 93 | 1 |
| CHEMBL329583 | Cc1cc2c(OC[C@H](O)CN3CC[C@@H](c4ccc5occc5c4)C[C@@H]3C)cccc2[nH]1 | 9 | 0 |
| CHEMBL327227 | O[C@@H](COc1cccc2[nH]ccc12)CN1CCC(c2cc3ccccc3s2)CC1 | 12 | 0 |
| CHEMBL314163 | Cc1cc2c(OC[C@H](O)CN3CC[C@@H](c4ccc5sccc5c4)C[C@@H]3C)cccc2[nH]1 | 12 | 0 |
| CHEMBL313023 | Cc1cc2c(OC[C@H](O)CN3CC[C@@H](c4cccc5sccc45)C[C@@H]3C)cccc2[nH]1 | 15 | 0 |
| CHEMBL301677 | O[C@H](COc1cccc2[nH]ccc12)CN1CCC(c2cc3c(F)cccc3s2)CC1 | 11.17 | 0 |
| CHEMBL301272 | Oc1cccc2cc(C3CCN(C[C@H](O)COc4cccc5[nH]ccc45)CC3)sc12 | 12.52 | 0 |
| CHEMBL300340 | COc1cccc2sc(C3CCN(C[C@H](O)COc4cccc5[nH]c(C)cc45)CC3)cc12 | 9.05 | 0 |
| CHEMBL297742 | Cc1cc2c(OC[C@@H](O)CN3CCC(c4cc5ccc(F)cc5s4)CC3)cccc2[nH]1 | 12.61 | 0 |
| CHEMBL294880 | Cc1cc2c(OC[C@@H](O)CN3CCC(c4cc5c(C)cccc5s4)CC3)cccc2[nH]1 | 12.09 | 0 |
| CHEMBL293065 | Cc1cc2c(OC[C@@H](O)CN3CCC(c4cc5cc(F)ccc5s4)CC3)cccc2[nH]1 | 12.06 | 0 |
| CHEMBL286003 | COc1ccccc1OCCNCC1COC(c2ccccc2)(c2ccccc2)O1 | 31.73333 | 0 |
| CHEMBL285157 | C1=C(c2c[nH]c3ccccc23)CCN(Cc2ccccc2)C1 | 89 | 1 |
| CHEMBL2431282 | CCCc1ccccc1OC(C)C1=NCCN1 | 118 | 1 |
| CHEMBL2413154 | COc1ccccc1N1CCN(CCCCn2ncc(=O)n(C)c2=O)CC1 | 80 | 1 |
| CHEMBL2413153 | Cn1c(=O)cnn(CCCCN2CCN(c3ccccc3OCCF)CC2)c1=O | 77 | 1 |
| CHEMBL236518 | OC(CCNC1CCCCC1)COc1cccc2[nH]ccc12 | 45.6 | 0 |
| CHEMBL234853 | Fc1ccc2[nH]cc(CCCNC3COc4ccc5ncccc5c4C3)c2c1 | 93 | 1 |
| CHEMBL231471 | O=C(c1ccc(F)c(Cl)c1)N1CCC(F)(CNCc2nccc(-c3ccco3)n2)CC1 | 79 | 1 |
| CHEMBL231373 | CNc1ccnc(CNCC2(F)CCN(C(=O)c3ccc(F)c(Cl)c3)CC2)n1 | 58.8 | 1 |
| CHEMBL231372 | COc1ccnc(CNCC2(F)CCN(C(=O)c3ccc(F)c(Cl)c3)CC2)n1 | 75.5 | 1 |
| CHEMBL231273 | O=C(c1ccc(F)c(Cl)c1)N1CCC(F)(CNCc2ncc(Cl)cn2)CC1 | 76 | 1 |
| CHEMBL231272 | O=C(c1ccc(F)c(Cl)c1)N1CCC(F)(CNCc2ncc(C(F)F)cn2)CC1 | 82.5 | 1 |
| CHEMBL231068 | O=c1[nH]c2ccccc2n1CCN1CCN(c2cccc(C(F)(F)F)c2)CC1 | 72 | 1 |
| CHEMBL230961 | Cc1ccc(CNCC2(F)CCN(C(=O)c3ccc(F)c(Cl)c3)CC2)nn1 | 89.7 | 1 |
| CHEMBL230425 | O=C(c1ccc(F)c(Cl)c1)N1CCC(F)(CNCc2ccc(CO)cn2)CC1 | 80 | 1 |
| CHEMBL230327 | O=C(c1ccc(F)c(Cl)c1)N1CCC(F)(CNCc2ccc(C(F)F)cn2)CC1 | 96.4 | 1 |
| CHEMBL2234448 | COc1ccccc1N1CCN(CC2COC3(CCCCC3)O2)CC1 | 48 | 0 |
| CHEMBL221957 | CC(C)Oc1ccccc1OCCNCc1cccc(C2=CCCCCC2)c1 | 86.5 | 1 |
| CHEMBL221920 | C1=C(c2cncc(CNCCOc3cccc4c3OC3(CC3)C4)c2)CCC1 | 78.5 | 1 |
| CHEMBL221743 | CC(C)Oc1ccccc1OCCNCc1cccc(C2=CCCCC2)c1 | 69.8 | 1 |
| CHEMBL221692 | Fc1ccc(-c2cncc(CN3CCN(c4cccc5c4OCCO5)CC3)c2)cc1 | 45.3 | 0 |
| CHEMBL221183 | CC1(C)COc2c(OCCNCc3cccc(C4=CCCC4)c3)cccc21 | 72.4 | 1 |
| CHEMBL221131 | CCC1(C)Cc2cccc(OCCNCc3cccc(C4=CCCC4)c3)c2O1 | 77.7 | 1 |
| CHEMBL220809 | CC(C)Oc1ccccc1OCCNCc1cccc(C2=CCCC2)c1 | 54 | 1 |
| CHEMBL220808 | Fc1ccc(-c2cncc(CNC[C@H]3CCc4ccccc4O3)c2)cc1 | 66.5 | 1 |
| CHEMBL219486 | CC1(C)Oc2cccc(OCCNCc3cccc(C4=CCCC4)c3)c2O1 | 90.6 | 1 |
| CHEMBL218313 | C1=C(c2cccc(CNCCOc3cccc4c3OC3(CC3)C4)c2)CCC1 | 93.8 | 1 |
| CHEMBL218261 | CC1(C)Cc2cccc(OCCNCc3cccc(C4=CCCC4)c3)c2O1 | 85.6 | 1 |
| CHEMBL218217 | CC(C)Oc1ccccc1OCCNCc1cccc(-c2ccccc2)c1 | 73.9 | 1 |
| CHEMBL218166 | O=c1[nH]c2cccc(N3CCN(Cc4cccc(-c5ccccc5)c4)CC3)c2o1 | 60.8 | 1 |
| CHEMBL213673 | NC(=O)c1ccc(F)c2c1CC(N(CCCCc1c[nH]c3ccc(F)cc13)C1CCC1)CO2 | 0 | 0 |
| CHEMBL214009 | CCCN(CCCc1c[nH]c2ccc(F)cc12)C1COc2c(F)ccc(C(N)=O)c2C1 | 81 | 1 |
| CHEMBL213639 | CCCN(CCc1c[nH]c2ccc(F)cc12)C1COc2c(F)ccc(C(N)=O)c2C1 | 0 | 0 |
| CHEMBL211480 | COc1cccc2c1CC(N(CCCc1c[nH]c3ccc(F)cc13)CC1CC1)CO2 | 91 | 1 |
| CHEMBL386288 | COc1cccc2c1C[C@@H](N(CCCc1c[nH]c3ccc(F)cc13)C1CCC1)CO2 | 0 | 0 |
| CHEMBL2113030 | C1=C(c2c[nH]c3ccccc23)CC2CCC1N2CCOc1cccc2c1OCCO2 | 65 | 1 |
| CHEMBL2112779 | COc1cccc(C2CCC(N3CCN(c4ccccc4OC)CC3)CC2)c1 | 26.5 | 0 |
| CHEMBL511857 | COc1cccc(C2CCC(N3CCN(c4ccccn4)CC3)CC2)c1 | 95.9 | 1 |
| CHEMBL2112774 | COc1cccc(C2CCC(NCCOc3ccccn3)CC2)c1 | 82 | 1 |
| CHEMBL2112358 | Cc1cc2c(OC[C@@H](O)CN3CC[C@@H](c4ccc5ccsc5c4)C[C@@H]3C)cccc2[nH]1 | 12 | 0 |
| CHEMBL2112357 | Cc1cc2c(OC[C@@H](O)CN3CC[C@@H](c4cccc5ccsc45)C[C@@H]3C)cccc2[nH]1 | 8 | 0 |
| CHEMBL2112356 | Cc1cc2c(OC[C@@H](O)CN3CC[C@@H](c4ccc5scc(C)c5c4)C[C@@H]3C)cccc2[nH]1 | 8 | 0 |
| CHEMBL2112355 | Cc1cc2c(OC[C@@H](O)CN3CC[C@@H](c4ccc5c(C)csc5c4)C[C@@H]3C)cccc2[nH]1 | 4 | 0 |
| CHEMBL2112354 | Cc1cc2c(OC[C@@H](O)CN3CC[C@@H](c4ccc5ccoc5c4)C[C@@H]3C)cccc2[nH]1 | 6 | 0 |
| CHEMBL2112353 | COc1cccc2ccc([C@@H]3CCN(C[C@H](O)COc4cccc5[nH]c(C)cc45)[C@@H](C)C3)cc12 | 11 | 0 |
| CHEMBL2112352 | Cc1cc2c(OC[C@@H](O)CN3CC[C@@H](c4cccc5occc45)C[C@@H]3C)cccc2[nH]1 | 8 | 0 |
| CHEMBL2112350 | Cc1cc2c(OC[C@@H](O)CN3CC[C@@H](c4ccc5ccccc5c4)C[C@@H]3C)cccc2[nH]1 | 9 | 0 |
| CHEMBL211393 | CCN(CCCc1c[nH]c2ccc(F)cc12)[C@@H]1COc2c(F)ccc(C(N)=O)c2C1 | 91 | 1 |
| CHEMBL427051 | NC(=O)c1ccc(F)c2c1C[C@H](N(CCCc1c[nH]c3ccc(F)cc13)CC1CC1)CO2 | 69 | 1 |
| CHEMBL210656 | COc1cccc2c1CC(N(C)CCCc1c[nH]c3ccc(F)cc13)CO2 | 91 | 1 |
| CHEMBL210348 | CCN(CCc1c[nH]c2ccc(F)cc12)C1COc2c(F)ccc(C(N)=O)c2C1 | 0 | 0 |
| CHEMBL209821 | CC(=O)Nc1cccc(N2CCN(CCCCNS(=O)(=O)CC3CCCCC3)CC2)c1 | 6.6 | 0 |
| CHEMBL209784 | CCCN(CCCCc1c[nH]c2ccc(F)cc12)C1COc2c(F)ccc(C(N)=O)c2C1 | 0 | 0 |
| CHEMBL209262 | CCN(CCCc1c[nH]c2ccc(F)cc12)C1COc2cccc(OC)c2C1 | 96 | 1 |
| CHEMBL2089156 | C[C@@H](Oc1ccccc1CC1CC1)C1=NCCN1 | 68 | 1 |
| CHEMBL208773 | NC(=O)c1ccc(F)c2c1C[C@@H](N(CCCc1c[nH]c3ccc(F)cc13)C1CCC1)CO2 | 0 | 0 |
| CHEMBL208605 | CCN(CCCCc1c[nH]c2ccc(F)cc12)C1COc2c(F)ccc(C(N)=O)c2C1 | 0 | 0 |
| CHEMBL206644 | CCN1CC(CNCCc2c[nH]c3ccc(F)cc23)Oc2ccccc21 | 100 | 1 |
| CHEMBL206130 | COc1cccc2c1OC(CNCCc1c[nH]c3ccc(F)cc13)CN2 | 66 | 1 |
| CHEMBL205031 | C1=C(c2c[nH]c3ccccc23)CCN(CC2CNc3ccccc3O2)C1 | 62 | 1 |
| CHEMBL204331 | CC(C)N1CC(CNCCCc2c[nH]c3ccccc23)Oc2ccccc21 | 88 | 1 |
| CHEMBL203726 | CCN1CC(CN2CC=C(c3c[nH]c4ccc(F)cc34)CC2)Oc2c(OC)cccc21 | 54 | 1 |
| CHEMBL203282 | CC(C)CN1CC(CNCCc2c[nH]c3ccc(F)cc23)Oc2ccccc21 | 88 | 1 |
| CHEMBL203067 | CN1CC(CNCCc2c[nH]c3ccccc23)Oc2ccccc21 | 95 | 1 |
| CHEMBL203018 | COc1cccc2c1OC(CN1CC=C(c3c[nH]c4ccc(F)cc34)CC1)CN2C | 93 | 1 |
| CHEMBL200951 | CN1CCc2c(sc3ncn(CCN4CCN(c5ccc6ccccc6n5)CC4)c(=O)c23)C1 | 10 | 0 |
| CHEMBL200950 | COc1ccc2ccccc2c1N1CCN(CCn2cnc3sc4c(c3c2=O)CCN(C)C4)CC1 | 10 | 0 |
| CHEMBL200315 | CN1CCc2c(sc3ncn(CCN4CCN(c5cccc6c5OCC6)CC4)c(=O)c23)C1 | 10 | 0 |
| CHEMBL199443 | CN1CCc2c(sc3ncn(CCN4CCN(c5cccc6cccnc56)CC4)c(=O)c23)C1 | 10 | 0 |
| CHEMBL199269 | CN1CCc2c(sc3ncn(CCN4CCN(c5cccc6cnccc56)CC4)c(=O)c23)C1 | 10 | 0 |
| CHEMBL199049 | COc1ccccc1N1CCN(CCn2cnc3sc4c(c3c2=O)CCN(C)C4)CC1 | 10 | 0 |
| CHEMBL198999 | CN1CCc2c(sc3ncn(CCN4CCN(c5ccccc5OCc5ccccc5)CC4)c(=O)c23)C1 | 10 | 0 |
| CHEMBL198836 | CN1CCc2c(sc3ncn(CCN4CCN(c5cccc6ccccc56)CC4)c(=O)c23)C1 | 10 | 0 |
| CHEMBL198792 | CN1CCc2c(sc3ncn(CCN4CCN(c5ccccc5Oc5ccccc5)CC4)c(=O)c23)C1 | 10 | 0 |
| CHEMBL198696 | CN1CCc2c(sc3ncn(CCN4CCN(c5ccccc5Cl)CC4)c(=O)c23)C1 | 10 | 0 |
| CHEMBL198463 | CN1CCc2c(sc3ncn(CCN4CCN(c5ccnc6ccccc56)CC4)c(=O)c23)C1 | 6 | 0 |
| CHEMBL198462 | COc1ccc(Cl)cc1N1CCN(CCn2cnc3sc4c(c3c2=O)CCN(C)C4)CC1 | 10 | 0 |
| CHEMBL197445 | COc1c(Cl)cc(Cl)cc1N1CCN(CCn2cnc3sc4c(c3c2=O)CCN(C)C4)CC1 | 70 | 1 |
| CHEMBL197344 | CN1CCc2c(sc3ncn(CCN4CCN(c5cccc6ccsc56)CC4)c(=O)c23)C1 | 10 | 0 |
| CHEMBL197338 | COc1cc(Cl)c(Cl)cc1N1CCN(CCn2cnc3sc4c(c3c2=O)CCN(C)C4)CC1 | 10 | 0 |
| CHEMBL197240 | CN1CCc2c(sc3ncn(CCN4CCN(c5cccc6ncccc56)CC4)c(=O)c23)C1 | 10 | 0 |
| CHEMBL197078 | Cc1ccccc1N1CCN(CCn2cnc3sc4c(c3c2=O)CCN(C)C4)CC1 | 10 | 0 |
| CHEMBL197064 | COc1ccc(C)cc1N1CCN(CCn2cnc3sc4c(c3c2=O)CCN(C)C4)CC1 | 10 | 0 |
| CHEMBL197011 | COc1ccc(OC)c(N2CCN(CCn3cnc4sc5c(c4c3=O)CCN(C)C5)CC2)c1 | 10 | 0 |
| CHEMBL197007 | CN1CCc2c(sc3ncn(CCN4CCN(c5ccccc5-c5ccccc5)CC4)c(=O)c23)C1 | 10 | 0 |
| CHEMBL196962 | COc1cc(Cl)ccc1N1CCN(CCn2cnc3sc4c(c3c2=O)CCN(C)C4)CC1 | 10 | 0 |
| CHEMBL196958 | CC(C)Oc1ccccc1N1CCN(CCn2cnc3sc4c(c3c2=O)CCN(C)C4)CC1 | 10 | 0 |
| CHEMBL196914 | COc1c(Cl)cccc1N1CCN(CCn2cnc3sc4c(c3c2=O)CCN(C)C4)CC1 | 10 | 0 |
| CHEMBL196907 | CN1CCc2c(sc3ncn(CCN4CCN(c5ncnc6ccccc56)CC4)c(=O)c23)C1 | 10 | 0 |
| CHEMBL196862 | CN1CCc2c(sc3ncn(CCN4CCN(c5ccccc5C#N)CC4)c(=O)c23)C1 | 10 | 0 |
| CHEMBL1946788 | COc1ccccc1N1CCN(C[C@H]2CCC(c3ccccc3)(c3ccccc3)[C@@H]2O)CC1 | 37.3 | 0 |
| CHEMBL1946786 | COc1ccccc1OCCNC[C@H]1CCC(c2ccccc2)(c2ccccc2)[C@@H]1O | 64.1 | 1 |
| CHEMBL1946785 | O[C@@H]1[C@@H](CNCCOc2ccccc2)CCC1(c1ccccc1)c1ccccc1 | 64.9 | 1 |
| CHEMBL1946782 | COc1ccccc1OCCNCC1CCC(c2ccccc2)(c2ccccc2)C1=O | 39.4 | 0 |
| CHEMBL1946781 | O=C1C(CNCCOc2ccccc2)CCC1(c1ccccc1)c1ccccc1 | 25.5 | 0 |
| CHEMBL1946779 | COc1ccccc1OCCNCC1CCC(c2ccccc2)(c2ccccc2)O1 | 53.4 | 1 |
| CHEMBL1946777 | COc1ccccc1N1CCN(CC2COC(c3ccccc3)(c3ccccc3)O2)CC1 | 16.2 | 0 |
| CHEMBL193206 | C[C@H](CN(C(=O)c1ccc([N+](=O)[O-])cc1)c1ccccn1)N1CCN(c2cccc3c2OCCO3)CC1 | 0 | 0 |
| CHEMBL1926761 | O=C1c2ccccc2S(=O)(=O)N1CCCCNCC1CCc2ccccc2O1 | 94.8 | 1 |
| CHEMBL1926760 | O=C1CSC(=O)N1CCCCCCCCNCC1CCc2ccccc2O1 | 88.6 | 1 |
| CHEMBL1926759 | O=C1CSC(=O)N1CCCCCCNCC1CCc2ccccc2O1 | 87 | 1 |
| CHEMBL1926758 | O=C1CSC(=O)N1CCCCCNCC1CCc2ccccc2O1 | 94.8 | 1 |
| CHEMBL1926751 | O=C1C2CCCN2C(=O)N1CCCCCCNCC1CCc2ccccc2O1 | 91.2 | 1 |
| CHEMBL1926750 | O=C1C2CCCN2C(=O)N1CCCNCC1CCc2ccccc2O1 | 94.3 | 1 |
| CHEMBL1926749 | O=C1CSC(=O)N1CCCCNCC1CCc2ccccc2O1 | 89.6 | 1 |
| CHEMBL1926748 | O=C1C2CSCN2C(=O)N1CCCCNCC1CCc2ccccc2O1 | 89.3 | 1 |
| CHEMBL1926747 | O=C1C2CCSN2C(=O)N1CCCCNCC1CCc2ccccc2O1 | 94.5 | 1 |
| CHEMBL1926745 | O=C1C2CCCN2C(=O)CN1CCCCNCC1CCc2ccccc2O1 | 87.6 | 1 |
| CHEMBL1926744 | O=C1C2CCCCN2C(=O)N1CCCCNCC1CCc2ccccc2O1 | 77.9 | 1 |
| CHEMBL1926733 | O=C1C2CCCN2C(=O)N1CCCCNCC1CCc2ccccc2O1 | 94.6 | 1 |
| CHEMBL1926732 | O=C1C2CCCN2C(=O)N1CCCCNCc1ccc2ccccc2n1 | 75.8 | 1 |
| CHEMBL1926686 | O=C1C2CCCN2C(=O)N1CCCCCNCC1CCc2ccccc2O1 | 92.5 | 1 |
| CHEMBL18840 | NCCc1c[nH]c2ccc(C(N)=O)cc12 | 95 | 1 |
| CHEMBL187745 | C1=C(c2ccc3ccccc3c2)CC2CCC1N2CCOc1cccc2cccnc12 | 95 | 1 |
| CHEMBL187329 | C1=C(c2ccc3ccccc3c2)CC2CCC1N2CCOc1cccc2ncccc12 | 29 | 0 |
| CHEMBL186202 | O[C@H](COc1cccc2[nH]ccc12)CN1C2C=C(c3ccc4ccccc4c3)CC1CC2 | 0 | 0 |
| CHEMBL186140 | Clc1ccc(C2=CC3CCC(C2)N3CCOc2cccc3[nH]ccc23)cc1Cl | 5 | 0 |
| CHEMBL185624 | C1=C(c2ccc3ccccc3c2)CC2CCC1N2CCOc1cccc2[nH]ccc12 | 25 | 0 |
| CHEMBL185494 | C1=C(c2ccc3ccccc3c2)CC2CCC1N2CCCOc1cccc2[nH]ccc12 | 81 | 1 |
| CHEMBL181506 | COc1ccccc1N1CCN([C@H]2CC[C@@H](c3c[nH]c4ccc(F)cc43)CC2)CC1 | 5 | 0 |
| CHEMBL179648 | c1ccc(CN2CCN(CC3CCc4ccccc4O3)CC2)cc1 | 41 | 0 |
| CHEMBL178700 | c1ccc(CC2CCN(CC3COc4ccccc4O3)CC2)cc1 | 41.7 | 0 |
| CHEMBL178650 | c1ccc(CC2CCN(CC3CCc4ccccc4O3)CC2)cc1 | 61.9 | 1 |
| CHEMBL362823 | Fc1ccc2[nH]cc([C@H]3CC[C@@H](NCCOc4cccc5c4OCCO5)CC3)c2c1 | 27 | 0 |
| CHEMBL151266 | COc1cccc2c1O[C@@H](CN1[C@@H]3CC[C@H]1C[C@](O)(c1ccccc1)C3)CO2 | 0 | 0 |
| CHEMBL148860 | COc1cccc2c1O[C@@H](CN1[C@@H]3CC[C@H]1C[C@](O)(c1csc4ccccc14)C3)CO2 | 0 | 0 |
| CHEMBL146751 | COc1cccc2c1O[C@@H](CN1[C@@H]3CC[C@H]1C[C@](O)(c1ccc4ccccc4c1)C3)CO2 | 0 | 0 |
| CHEMBL1457510 | COc1c(O)ccc2c1CN1CCc3cc4c(cc3[C@@H]1C2)OCO4 | 80.7 | 1 |
| CHEMBL138109 | COc1cccc2c(CCCNCCOc3ccccn3)cccc12 | 48 | 0 |
| CHEMBL131370 | Cc1cc2c(OC[C@@H](O)CN3CC[C@@H](c4cc5c(C)cccc5s4)C[C@@H]3C)cccc2[nH]1 | 6.32 | 0 |
| CHEMBL129682 | COc1cccc2sc([C@@H]3C[C@H]4CC[C@@H](C3)N4C[C@H](O)COc3cccc4[nH]c(C(N)=O)cc34)cc12 | 11.26 | 0 |
| CHEMBL129650 | COc1cccc2sc([C@@H]3CCN(C[C@H](O)COc4cccc5oc(C)cc45)[C@@H](C)C3)cc12 | 8.76 | 0 |
| CHEMBL129233 | COc1cccc2sc([C@@H]3C[C@H]4CC[C@@H](C3)N4C[C@H](O)COc3cccc4[nH]c(C)cc34)cc12 | 2.98 | 0 |
| CHEMBL129433 | COc1cccc2sc([C@@H]3CCN(C[C@H](O)COc4cccc5[nH]c(C(N)=O)cc45)[C@@H](C)C3)cc12 | 12.75 | 0 |
| CHEMBL129238 | Cc1cc2c(OC[C@@H](O)CN3CC[C@@H](c4cc5cc(F)ccc5s4)C[C@@H]3C)cccc2[nH]1 | 9.05 | 0 |
| CHEMBL129082 | Cc1cc2c(OC[C@@H](O)CN3CC[C@@H](c4cc5c(F)cccc5s4)C[C@@H]3C)cccc2[nH]1 | 3.15 | 0 |
| CHEMBL129053 | COc1cccc2sc([C@@H]3CCN(C[C@H](O)COc4cccc5[nH]c(C)cc45)[C@@H](C)C3)cc12 | 3.88 | 0 |
| CHEMBL128866 | COc1cc(OC)c2cc([C@@H]3CCN(C[C@H](O)COc4cccc5[nH]c(C)cc45)[C@@H](C)C3)sc2c1 | 7.62 | 0 |
| CHEMBL1278088 | Nn1c(CCCCN2CCN(c3ccc4ccccc4n3)CC2)nc2c(c1=O)CCCC2 | 99.8 | 1 |
| CHEMBL1277651 | Nn1c(CCCCN2CCN(c3ccc4ccccc4n3)CC2)nc2ccccc2c1=O | 94.7 | 1 |
| CHEMBL1277558 | CC(=O)N1CCc2c(sc3nc(SCCCN4CCN(c5ccc6ccccc6n5)CC4)n(N)c(=O)c23)C1 | 84.8 | 1 |
| CHEMBL1277281 | Nn1c(SCCCN2CCN(c3ccc4ccccc4n3)CC2)nc2c(c1=O)CCCC2 | 90.3 | 1 |
| CHEMBL1277007 | Cc1cc(N2CCN(CCCCc3nc4c(c(=O)n3N)CCCC4)CC2)nc2ccccc12 | 93.5 | 1 |
| CHEMBL1276220 | C[C@H](Oc1ccccc1C1CC1)C1=NCCN1 | 102 | 1 |
| CHEMBL495095 | C=CCc1ccccc1O[C@@H](C)C1=NCCN1 | 96 | 1 |
| CHEMBL1259242 | Cc1ccc(CNCC2(F)CCN(C(=O)c3cc4ccccc4s3)CC2)nc1 | 116 | 1 |
| CHEMBL1259241 | Cc1ccc(CNCC2(F)CCN(C(=O)c3csc4ccccc34)CC2)nc1 | 124 | 1 |
| CHEMBL1259224 | Cc1ccc(CNCC2(F)CCN(C(=O)C34CC5CC(CC(Cl)(C5)C3)C4)CC2)nc1 | 91 | 1 |
| CHEMBL1259223 | Cc1ccc(CNCC2(F)CCN(C(=O)C34CC5CC(CC3C5)C4)CC2)nc1 | 96 | 1 |
| CHEMBL1259210 | Cc1ccc(CNCC2(F)CCN(C(=O)C34CCCC(CCC3)C4)CC2)nc1 | 96 | 1 |
| CHEMBL1259209 | Cc1ccc(CNCC2(F)CCN(C(=O)C34CC5CC(CC(C5)C3)C4)CC2)nc1 | 98 | 1 |
| CHEMBL1259200 | Cc1ccc(CNCC2(O)CC3CCC(C2)N3C(=O)c2ccc(F)c(Cl)c2)nc1 | 121 | 1 |
| CHEMBL1259198 | COc1c(C(=O)N2CCC(F)(CNCc3ccc(C)cn3)CC2)csc1Cl | 102 | 1 |
| CHEMBL1259184 | Cc1ccc(CNCC2(O)CC3CCC(C2)N3C(=O)c2ccsc2)nc1 | 101 | 1 |
| CHEMBL1259181 | Cc1ccc(CNCC2(F)CCN(C(=O)c3ccc4ccccc4c3)CC2)nc1 | 110 | 1 |
| CHEMBL1259165 | COc1cscc1C(=O)N1CCC(F)(CNCc2ccc(C)cn2)CC1 | 93 | 1 |
| CHEMBL1259155 | Cc1ccc(CNCC2(O)CCN(C(=O)c3ccc(F)c(Cl)c3)CC2)nc1 | 109 | 1 |
| CHEMBL1259153 | CSc1ccc(C(=O)N2CCC(F)(CNCc3ccc(C)cn3)CC2)s1 | 91 | 1 |
| CHEMBL1259151 | Cc1ccc(CNCC2(F)CCN(C(=O)c3ccccc3)CC2)nc1 | 96 | 1 |
| CHEMBL1259139 | Cc1ccc(CNCC2(F)CCN(C(=O)c3sccc3Cl)CC2)nc1 | 99 | 1 |
| CHEMBL1259138 | Cc1ccc(CNCC2(F)CCN(C(=O)c3ccc(Cl)s3)CC2)nc1 | 101 | 1 |
| CHEMBL1259124 | Cc1ccc(CN(C)CC2(F)CCN(C(=O)c3ccc(F)c(Cl)c3)CC2)nc1 | 77 | 1 |
| CHEMBL1259123 | Cc1ccc(CNCC2(F)CCN(C(=O)c3sc4sccc4c3Cl)CC2)nc1 | 97 | 1 |
| CHEMBL1259122 | Cc1ccc(CNCC2(F)CCN(C(=O)c3sccc3C)CC2)nc1 | 96 | 1 |
| CHEMBL1259121 | Cc1ccc(CNCC2(F)CCN(C(=O)c3ccc(C)s3)CC2)nc1 | 104 | 1 |
| CHEMBL1259109 | Cc1ccc(CNCC2(F)CCN(C(=O)c3cc4ccsc4s3)CC2)nc1 | 103 | 1 |
| CHEMBL1259108 | Cc1ccc(CNCC2(F)CCN(C(=O)c3cc4sccc4s3)CC2)nc1 | 100 | 1 |
| CHEMBL1259107 | Cc1ccc(CNCC2(F)CCN(C(=O)c3cc(C)cs3)CC2)nc1 | 101 | 1 |
| CHEMBL1259090 | Cc1ccc(CNCC2(F)CCN(C(=O)c3sc(Br)c4c3OCCO4)CC2)nc1 | 102 | 1 |
| CHEMBL1259089 | Cc1ccc(CNCC2(F)CCN(C(=O)c3scc4c3CCCC4)CC2)nc1 | 104 | 1 |
| CHEMBL1259087 | Cc1ccc(CNCC2(F)CCN(C(=O)c3sccc3Br)CC2)nc1 | 104 | 1 |
| CHEMBL1259072 | Cc1ccc(CNCC2(F)CCN(C(=O)c3ccc(Br)s3)CC2)nc1 | 95 | 1 |
| CHEMBL1259071 | Cc1ccc(CNCC2(F)CCN(C(=O)c3cc(Br)cs3)CC2)nc1 | 116 | 1 |
| CHEMBL1259055 | Cc1ccc(CNCC2(F)CCN(C(=O)c3ccc4[nH]ccc4c3)CC2)nc1 | 94 | 1 |
| CHEMBL1259051 | Cc1ccc(CNCC2(F)CCN(C(=O)c3ccoc3)CC2)nc1 | 99 | 1 |
| CHEMBL1259036 | Cc1ccc(CNCC2(F)CCN(C(=O)c3cc4ccccc4[te]3)CC2)nc1 | 46 | 0 |
| CHEMBL1259035 | Cc1ccc(CNCC2(F)CCN(C(=O)c3cc4ccccn4n3)CC2)nc1 | 80 | 1 |
| CHEMBL1259034 | Cc1ccc(CNCC2(F)CCN(C(=O)c3ccco3)CC2)nc1 | 81 | 1 |
| CHEMBL1259033 | Cc1ccc(CNCC2(F)CCN(C(=O)c3ccsc3)CC2)nc1 | 95 | 1 |
| CHEMBL1259032 | Cc1ccc(CNCC2(F)CCN(C(=O)c3cccs3)CC2)nc1 | 91 | 1 |
| CHEMBL1259017 | Cc1ccc(CNCC2(F)CCN(C(=O)c3cnn4ccccc34)CC2)nc1 | 96 | 1 |
| CHEMBL1259016 | Cc1ccc(CNCC2(F)CCN(C(=O)c3cc4ccccc4o3)CC2)nc1 | 87 | 1 |
| CHEMBL1258999 | Cc1ccc(CNCC2(F)CCN(C(=O)c3coc4ccccc34)CC2)nc1 | 101 | 1 |
| CHEMBL1258996 | Cc1ccc(CNCC2(F)CCN(C(=O)C34CC5CC(CC(O)(C5)C3)C4)CC2)nc1 | 90 | 1 |
| CHEMBL1255610 | COc1c(C(=O)N2CCC(F)(CNCc3ccc(C)cn3)CC2)csc1Br | 95 | 1 |
| CHEMBL1242818 | COc1ccccc1N1CCN(C[C@H]2CO[C@@](CN3C(=O)CCCC3=O)(c3ccccc3)O2)CC1 | 34 | 0 |
| CHEMBL1242724 | COc1ccccc1N1CCN(C[C@H]2CO[C@@](CN3C(=O)CCC3=O)(c3ccccc3)O2)CC1 | 45 | 0 |
| CHEMBL1242633 | COc1ccccc1N1CCN(C[C@@H]2CO[C@@](CN3CCCCCC3=O)(c3ccccc3)O2)CC1 | 39 | 0 |
| CHEMBL1242445 | COc1ccccc1N1CCN(C[C@@H]2CO[C@](CN3CCCC3=O)(c3ccccc3)O2)CC1 | 34 | 0 |
| CHEMBL1242352 | COc1ccccc1N1CCN(CC2OCC(c3ccccc3)(c3ccccc3)O2)CC1 | 16 | 0 |
| CHEMBL1241557 | COc1ccccc1N1CCN(C[C@@H]2CO[C@@](CN3C(=O)CC4(CCCC4)CC3=O)(c3ccccc3)O2)CC1 | 60 | 1 |
| CHEMBL1088072 | COc1ccccc1CCCNCC1COC(c2ccccc2)(c2ccccc2)O1 | 14 | 0 |
| CHEMBL1086156 | COc1ccccc1OCCNCC1CSC(c2ccccc2)(c2ccccc2)S1 | 20 | 0 |
| CHEMBL1083182 | COc1ccccc1OCCNCC1CSC(c2ccccc2)(c2ccccc2)O1 | 76 | 1 |
| CHEMBL1078953 | CCOc1ccccc1OCCNCC1COC(c2ccccc2)(c2ccccc2)O1 | 36 | 0 |
| CHEMBL1078773 | CC(C)Oc1ccccc1OCCNCC1COC(c2ccccc2)(c2ccccc2)O1 | 25 | 0 |
| CHEMBL1078772 | c1ccc(-c2ccccc2OCCNCC2COC(c3ccccc3)(c3ccccc3)O2)cc1 | 73 | 1 |
| CHEMBL1077168 | COc1cccc(OCCNCC2COC(c3ccccc3)(c3ccccc3)O2)c1 | 14 | 0 |

**Table S2: Processed I-5HT1A set**

| SMILES | Label |
| --- | --- |
| O=c1ccc2c([nH]1)c(c[nH]2)C1=CCNCC1 | 1 |
| C=CCN1C[C@@H](C[C@H]2[C@H]1Cc1c[nH]c3c1c2ccc3)C(=O)N(C(=O)NCC)CCCN(C)C | 1 |
| CCCCCCOc1nsnc1C1=CCCN(C1)C | 1 |
| CCCN([C@@H]1CCc2c(C1)c1cc([nH]c1cc2)C#N)CCC | 1 |
| CC(C[C@H]1C(=O)N2CCC[C@H]2[C@]2(N1C(=O)[C@@](O2)(NC(=O)[C@H]1CN(C)[C@H]2C(=C1)c1cccc3c1c(C2)c([nH]3)Br)C(C)C)O)C | 1 |
| CCCN([C@H]1Cc2c[nH]c3c2c(C1)c(cc3)C(=O)C)CCC | 1 |
| CCCN1C[C@H](CSC)C[C@H]2[C@H]1Cc1c[nH]c3c1c2ccc3 | 1 |
| CCc1[nH]c2c(c1CCN(C)C)cc(cc2)OC | 1 |
| CN(C1CCc2c(C1)c1cc(ccc1[nH]2)NC(=O)c1ccc(cc1)F)C | 1 |
| COc1ccc(cc1OCCN(C)C)NC(=O)c1ccc(cc1)c1ccc(cc1C)c1noc(n1)C | 1 |
| CN1CCN(CC1)C1=c2cc(sc2=Nc2c(N1)cccc2)C | 1 |
| NCCc1c[nH]c2c1cc(cc2)c1onc(n1)Cc1ccc(cc1)NS(=O)(=O)C | 1 |
| NCCc1c[nH]c2c1cc(OCC(=O)N1CCN(CC1)c1ccc(cc1)C#N)cc2 | 1 |
| CCN(C(=O)[C@H]1CN(C)[C@H]2C(=C1)c1cccc3c1c(C2)c[nH]3)CC | 1 |
| Nc1c(Cl)cc(c2c1OCCO2)c1nn(c(=O)o1)C1CCN(CC1)CCc1ccccc1 | 1 |
| CN(CCc1c[nH]c2c1cc(cc2)Cn1cncn1)C | 1 |
| CCN(C(=O)N[C@@H]1CN(C)[C@H]2C(=C1)c1cccc3c1c(C2)c[nH]3)CC | 1 |
| COc1ccc2c(c1)c(c[nH]2)C1=CCNCC1 | 1 |
| Clc1ccc2c(c1)[C@H]1CN(C[C@@H]1c1c(O2)cccc1)C | 1 |
| O=C1Nc2c(C1)cc(c(c2)Cl)CCN1CCN(CC1)c1nsc2c1cccc2 | 1 |
| CN1CCC[C@@H]1Cc1c[nH]c2c1cc(cc2)CCS(=O)(=O)c1ccccc1 | 1 |
| CNS(=O)(=O)Cc1ccc2c(c1)c(CCN(C)C)c[nH]2 | 1 |
| Fc1cccc2c1O[C@H]1CNC[C@@H]1O2 | 1 |
| CN1CCN(CC1)C1=Nc2cc(Cl)ccc2Nc2c1cccc2 | 1 |
| Oc1ccc2c(c1)c(CCCCN1CCC(=CC1)c1ccccc1)c[nH]2 | 1 |
| CCCCN1CCCC1CNC(=O)c1cc(C#N)c2c(c1OC)cccc2 | 1 |
| Fc1ccc2c(c1)onc2C1CCN(CC1)CCc1c(C)nc2n(c1=O)cccc2C | 1 |
| OC[C@@H](c1ccc(cc1)F)NC1CCN(CC1)CCCc1c[nH]c2c1cc(cc2)n1cnnc1 | 1 |
| COc1ccc(cc1N1CCN(CC1)C)NC(=O)c1ccc(cc1)c1ccc(cc1C)c1noc(n1)C | 1 |
| CN(CCc1c[nH]c2c1cc(C[C@H]1COC(=O)N1)cc2)C | 1 |
| Nc1ccc(cc1)CCN1CCN(CC1)c1cccc(c1)C(F)(F)F | 1 |
| Fc1ccc(cc1)C(CCCN1CCN(CC1)c1ncc(cn1)F)O | 1 |
| OCCOCCN1CCN(CC1)C1=Nc2ccccc2Sc2c1cccc2 | 1 |
| CSc1ccccc1N1CCN(CC1)CCCCCC(=O)NC1CCCc2c1cccc2 | 1 |
| O=c1[nH]c2c(n1CCN1CCN(CC1)c1cccc(c1)C(F)(F)F)cccc2 | 1 |
| CNS(=O)(=O)CCc1ccc2c(c1)c(c[nH]2)C1CCN(CC1)C | 1 |
| O=C(NC1CCCc2c1cccc2)CCCCCN1CCN(CC1)c1ccccc1c1ccccc1 | 1 |
| c1cnc(nc1)N1CCN(CC1)Cc1ccc2c(c1)OCO2 | 1 |
| Cc1ccc(c(c1)C)Sc1ccccc1N1CCNCC1 | 1 |
| N#Cc1ccc(cc1)CNC(=O)CCCCCN1CCN(CC1)c1ccccc1c1ccccc1 | 1 |
| COc1ccc2c(c1)c(ccc2)N1CCN(CC1)CCNC(=O)c1ccc(cc1)F | 1 |
| Clc1cccc(c1)N1CCN(CC1)CC(C(c1ccccc1)c1ccccc1)O | 1 |
| COc1ccc2c(c1)c(ccc2)N1CCN(CC1)CCNC(=O)c1cccs1 | 1 |
| O=C1N(CCCCN2CCN(CC2)c2ncccn2)C(=O)[C@@H]2[C@H]1[C@@H]1C=C[C@H]2[C@@H]2[C@H]1C=C2 | 1 |
| Fc1ccc(cc1)C(=O)CC1CCN(C1)CCOc1cccc2c1OCCO2 | 1 |
| O=C1CC2(CCCC2)CC(=O)N1CCCCN1CCN(CC1)c1ncccn1 | 1 |
| O=C1N(CCCCN2CCN(CC2)c2ncccn2)C(=O)[C@@H]2[C@H]1[C@H]1CC[C@@H]2C1 | 1 |
| COC(=O)c1cccnc1N1CCN(CC1)CCCC(c1ccc(cc1)F)c1ccc(cc1)F | 1 |
| CN1CCC(CC1)c1c[nH]c2c1cc(cc2)NC(=O)c1ccc(cc1)F | 1 |
| C1COc2c(O1)cccc2N1CCN(CC1)C1Cc2c(C1)cccc2 | 1 |
| O=C1c2ccccc2S(=O)(=O)N1CCCCN1CCN(CC1)c1ncccn1 | 1 |
| O=C1CCc2c(N1)cc(cc2)OCCCCN1CCN(CC1)c1cccc(c1Cl)Cl | 1 |
| O=C1NCN(C21CCN(CC2)CC1COc2c(O1)cccc2)c1ccccc1 | 1 |
| COc1ccccc1N1CCN(CC1)CCN1C(=O)CC2(CC1=O)CCCC2 | 1 |
| Cc1cnc(nc1)CNCC1(F)CCN(CC1)C(=O)c1ccc(c(c1)Cl)F | 1 |
| CN(CCOC1=Cc2ccccc2Sc2c1cc(Cl)cc2)C | 0 |
| CCCN([C@@H]1CCc2c(C1)ccc(c2)CS(=O)(=O)c1ccc(cc1)OC)CCC | 0 |
| NC(=O)c1c([3H])cc(c2c1C[C@H](CO2)N(C1CCC1)C1CCC1)F | 0 |
| NC(=O)c1ccc(c2c1C[C@H](CO2)N(C1CCC1)C1CCC1)F | 0 |
| CCCN([C@H]1CCc2c(C1)c(O)ccc2F)CCC | 0 |
| COc1ccc2c(c1)c(CCN(C)C)c([nH]2)c1ccccc1 | 0 |
| CN(CCCN1c2ccccc2Sc2c1cc(Cl)cc2)C | 0 |
| Fc1ccc(cc1)C(=C1CCN(CC1)CCc1c(C)nc2n(c1=O)ccs2)c1ccc(cc1)F | 0 |
| CN1CCCCC1CCN1c2ccccc2Sc2c1cc(cc2)S(=O)C | 0 |
| CSc1ccc2c(c1)N(CCC1CCCCN1C)c1c(S2)cccc1 | 0 |
| CCN1CCC[C@H]1CNC(=O)c1c(O)c(Cl)cc(c1OC)Cl | 0 |
| O[C@]1(CCN2[C@@H](C1)c1cccc3c1[C@@H](C2)c1ccccc1CC3)C(C)(C)C | 0 |
| CN1CCC(=C2c3ccsc3CCc3c2cccc3)CC1 | 0 |
| CN1CCN(CC1)c1cc2c(cc1Cl)CCN2C(=O)Nc1ccc(c2c1cccc2)c1ccncc1 | 0 |
| CSc1ccc2c(c1)C(Cc1c(S2)cccc1)N1CCN(CC1)C | 0 |
| O=C(c1ccc(cc1)F)CCCN1CCC(CC1)(N1CCCCC1)C(=O)N | 0 |
| N#Cc1ccc2c(c1)N(CC(CN(C)C)C)c1c(S2)cccc1 | 0 |
| Fc1ccc(cc1)C(c1ccc(cc1)F)CCCN1CCC(CC1)n1c(=O)[nH]c2c1cccc2 | 0 |
| COC(=O)[C@H]1[C@@H](O)CC[C@@H]2[C@@H]1C[C@@H]1N(C2)CCc2c1[nH]c1c2cccc1 | 0 |
| Clc1ccc2c(c1)c(cn2c1ccc(cc1)F)C1CCN(CC1)CCN1CCNC1=O | 0 |
| Fc1ccc2c(c1)onc2C1CCN(CC1)CCc1c(C)nc2n(c1=O)CCCC2 | 0 |
| OC1CCc2n(C1)c(=O)c(c(n2)C)CCN1CCC(CC1)c1noc2c1ccc(c2)F | 0 |
| Fc1ccc(cc1)C(=O)CCCN1CCC2(CC1)C(=O)NCN2c1ccccc1 | 0 |
| Fc1ccc(cc1)C(c1ccc(cc1)F)CCCN1CCC2(CC1)C(=O)NCN2c1ccccc1 | 0 |
| O=C1COc2c(N1)cc(cc2)CC1CCN(CC1)CCOc1cccc2c1ccc(n2)C | 0 |
| Fc1ccc(cc1)C(=O)C1CCN(CC1)CCn1c(=O)[nH]c2c(c1=O)cccc2 | 0 |
| O[C@@H](COc1cccc2c1SCCC2)CNC(C)(C)C | 0 |
| COc1ccc(cc1N1CCN(CC1)C)NC(=O)c1ccc(c(c1)C)c1ccncc1 | 0 |
| Fc1ccc(cc1)C(=O)CCCN1CCC(CC1)(O)c1ccc(cc1)Cl | 0 |
| FCC(NC[C@H](COc1cccc2c1c1ccccc1[nH]2)O)C | 0 |
| O=C1CC2(CCCC2)CC(=O)N1CCCCN1CCN(CC1)c1nsc2c1cccc2 | 0 |
| Cc1ccc2c(n1)cccc2OCCN1CCN(CC1)Cc1cccc2c1nccc2 | 0 |
| O[C@H](COc1cccc2c1cccc2)CNC(C)C | 0 |
| COc1cc(ccc1OCCCN1CCC(CC1)c1noc2c1ccc(c2)F)C(=O)C | 0 |
| COC(=O)c1[nH]c2c(c1)c(ccc2)N1CCN(CC1)CCCCN1C(=O)c2c(S1(=O)=O)cccc2 | 0 |
| OC(COc1cccc2c1cc[nH]2)CNC(C)C | 0 |
| COc1ccccc1N1CCN(CC1)CCN(C(=O)c1ccc(cc1)I)c1ccccn1 | 0 |
| COc1ccccc1N1CCN(CC1)CCN(c1ccccc1[N+](=O)[O-])C(=O)C1CCCCC1 | 0 |
